# Fresh fruit surface: A potential source for multidrug-resistant biofilm-forming *Enterococcus faecalis* having potential public health significance

**DOI:** 10.1371/journal.pone.0355410

**Published:** 2026-08-05

**Authors:** Rownak Jahan, Naeem Ahammed Ibrahim Fahim, Md Abdullah Evna Hasan, Md. Liton Rana, Md. Tabeer Hossain Antor, Samia Salam, Rony Ibne Masud, Dilruba Akter Jany, Zuhayr Bakhtiyar, Md. Saiful Islam, Muhammad Tofazzal Hossain, Md. Tanvir Rahman

**Affiliations:** 1 Department of Microbiology and Hygiene, Faculty of Veterinary Science, Bangladesh Agricultural University, Mymensingh, Bangladesh; 2 National Engineering Research Center of Industrial Wastewater Detoxication and Resource Recovery, Research Center for Eco-Environmental Sciences, Chinese Academy of Sciences, Beijing, China; 3 University of Chinese Academy of Sciences, Beijing, China; 4 Department of Animal Science, University of California - Davis, Davis, California, United States of America; Universidad Autonoma de Chihuahua, MEXICO

## Abstract

Fruits are important in the diet because of the nutrients, vitamins, and minerals they provide for a healthy life; however, they can also serve as a potential source of opportunistic pathogens, including enterococci. They are considered zoonotic pathogens that cause severe infections in humans. This work aimed to isolate *Enterococcus faecalis* from fruit surfaces and to detect its virulence genes, biofilm-forming ability, and antibiotic resistance patterns. A total of 76 samples of seven different types of fruits were collected and surface-washed, and used to isolate and identify *E. faecalis* by cultural and biochemical tests, followed by polymerase chain reaction (PCR), the disk diffusion method for antimicrobial susceptibility testing, a Congo Red Agar test for the determination of biofilm-forming ability, and PCR for virulence gene detection. Out of 76 samples, 47 (61.84%) fruit samples were found to be positive for *E. faecalis,* with the highest prevalence in oranges compared to the other six fruits. In the phenotypic antibiogram study, resistance was most frequently observed against commonly used antibiotics, including ampicillin (90.00%) and erythromycin (57.50%), while notable resistance was also detected against vancomycin (47.50%) and the last-resort antibiotic linezolid (47.50%). The ampicillin-resistance gene, *blaTEM,* was present in 65% of the isolates. Additionally, 60% of isolates exhibited multidrug resistance (MDR), with the MAR index ranging from 0.33 to 0.78. Key virulence genes (*agg, ace, gelE, fsrB*, and *pil*) and biofilm-forming capabilities were distributed among the isolates, indicating their potential for persistence and pathogenicity. These findings suggest that fruit surfaces harbor antibiotic-resistant *E. faecalis*; therefore, fruits should be thoroughly washed before eating and monitored using One Health strategies to reduce the risk to human health.

## Introduction

Enterococci are commensal bacteria found in the intestines of both humans and domestic animals. They may also be found in the environment, including soil, water, plants, insects, birds, and wild animals. Currently, they rank third among the organisms that are often found in nosocomial infections [1]. *Enterococcus faecalis* and *Enterococcus faecium* can cause bacteremia, infective endocarditis, urinary tract infections, and wound infections in humans [2]. Enterococci can also infect the central nervous system [3]. Approximately 80–90 percent of human enterococcal infections are caused by *E. faecalis*, while most of the remaining cases are attributable to *E. faecium*. Among the various types of antibiotic-resistant enterococci, vancomycin-resistant enterococci (VRE) are a major global public health concern [4].

A healthy diet should include fruits, as they are rich in vitamins, minerals, fiber, and antioxidants, which are essential for maintaining good health, supporting immune function, and aiding digestion. Considering the nutritional benefits of fruits, fruit consumption is increasing significantly day by day. However, fruit safety is most seriously threatened by microbial contamination, particularly foodborne pathogens, as large and diverse bacterial communities can be found in fresh fruits and vegetables [5]. Sometimes, fresh fruits can be a source of entry point if they are eaten without washing or proper cleaning, making bacterial contamination a major concern for consumers [6,7]. Climate change, excessive pesticide use, inadequate postharvest handling and cleanliness, and fruit contamination all raise the danger of foodborne illnesses, financial losses, and food insecurity [8]. Enteropathogenic bacteria on the edible peel of fruits also increase consumer health risks. More than 10 pathogenic organisms were isolated from fresh-cut fruits in China, highlighting the potential role of fruits in the dissemination of microorganisms among community members [9]. Additionally, when fruit vendors use contaminated water to clean and wash their fruit, enterococci can spread and cause foodborne infections [10].

The development and spread of antibiotic resistance in bacteria are well-known global health concerns. Recently observed increases in multidrug-resistant (MDR) microorganisms that cause infections are growing worldwide and becoming more severe in developing countries. Enterococci could act as reservoirs of antibiotic resistance and are becoming increasingly alarming [6,11]. Consuming fresh fruit can introduce or elevate the baseline level of antibiotic-resistant bacteria in the body, which might make it hard to fight against them with existing drugs [12]. Additionally, raw foods have been found to have antibiotic-resistant *E. faecalis*, which may be connected to irrigation water that is directly passed to people [13]. The presence of antibiotic resistance genes (ARGs) in pathogenic bacteria associated with fresh fruits and liquids also raises safety concerns. The lack of antibiotic targets, low-affinity targets, impermeability to some antibiotics, efflux pumps, and lack of uptake pathways for different antimicrobials are examples of *E. faecalis’s* inherent resistance mechanisms [14–16]. Due to the unique combination of intrinsic and acquired antibiotic tolerance, Esp and Ebp proteins enable *E. faecalis* to form modestly organized but extremely persistent biofilms, making its infections particularly challenging to treat [17]. It remains unknown how *E. faecalis* persists and adapts to diverse environments, despite recent research identifying new resistance elements, gene transfer events, and surface proteins. Meta-analyses have revealed that virulence genes (*esp, gelE*) and resistance genes (*vanA, ermB*) co-occur in clinical and environmental isolates, suggesting horizontal gene transfer [18–20].

To the best of our knowledge, no prior study has examined the antibiotic resistance, biofilm formation, and virulence factors of *E. faecalis* isolated from fresh fruit surfaces in Bangladesh, particularly in the Mymensingh region. In this study, we aimed to address these knowledge gaps and examine how fresh fruits may contribute to the transmission of *E. faecalis* and the spread of antibiotic resistance.

## Materials and methods

### Sample collection and preparation

Between May 2024 and September 2024, 76 samples from various fruit shops, located in Chorpara (24.7464°N, 90.4092°E), Sesh more (24.7178°N, 90.4442°E), Kewatkhali (24.7316°N, 90.4163°E), Wapda more(24.7460°N, 90.4179°E), Bridge more (24.7470°N, 90.4200°E), Mechua Bazar (24.7558°N, 90.4100°E), and Notun Bazar (24.7589°N, 90.4025°E) were collected around Mymensingh town (24.7460°N, 90.4179°E) in Bangladesh. The areas were chosen for the study, located near medical entrances throughout the town, including Bangladesh Agricultural University, and the samples were collected using random sampling. The study did not involve any human or animal subjects. These samples included 18 apples (*Malus domestica*), 10 oranges (*Citrus sinensis*),15 maltas (*Citrus reticulata*), 16 grapes (*Vitis vinifera*), 8 mangoes (*Mangifera indica*), 5 guavas (*Psidium guajava*), and 4 dates (*Phoenix dactylifera*). To maintain a cold chain, all fruits were placed in separate zip-lock bags with unique tag numbers and sent directly to the Bacteriology laboratory of the Department of Microbiology and Hygiene (24.7245° N, 90.4372° E). Each sample was washed with 10 mL PBS, and 100 µL of washed PBS was transferred to a sterile Eppendorf tube containing 1 mL of nutrient broth (HiMedia, India). The test tubes were then incubated aerobically overnight at 37°C to promote bacterial growth [21,22].

### Isolation and Molecular Identification of *E. faecalis*

The primary isolation of *E. faecalis* was performed by culture on an Enterococcus Agar Base (EAB) medium (HiMedia, Mumbai, Maharashtra, India). A loopful of inoculum from the enriched culture (~ 0.5 McFarland concentration) was streaked onto an EAB Petri dish and incubated at 37°C for 16–24 hours. A yellowish circular colony on EAB medium was considered as *E. faecalis* and further confirmed by Gram’s staining and multiple biochemical tests, including sugar fermentation test, Voges–Proskauer test, indole test, and catalase activity. Finally, *E. faecalis* was identified at the molecular level by targeting *ddl*_*E. faecalis*_ gene using simplex PCR as described by [23]. **(**Table 1).

**Table 1:** List of primers used for specific detection of E. faecalis isolates with their virulence and antibiotic resistance genes.

Target Organism	Target genes	Primer sequence (5′-3′)	Amplicon size	References
***E. faecalis***	*ddl*	F: ATCAAGTACAGTTAGTCTT R: ACGATTCAAAGCTAACTG	941 bp	[25]
**Virulence gene**	*gelE*	F: GGTGAAGAAGTTACTCTGAC R: GGTATTGAGTTATG*AGG*GGC	704 bp	[26]
	*agg*	F: TCTTGGACACGACCCATGAT R: AGAAAGAACATCACCACGAC	413 bp	
	*pil*	F: GAAGAAACCAAAGCACCTAC R: CTACCTAAGAAAAGAAACGG	620 bp	
	*fsrB*	F: TAATCT*AGG*CTTAGTTCCCAC R: CTAAATGGCTCTGTCGTCTAG	428 bp	
	*ace*	F: GAATGACCGAGAACGATGGC R: CTTGATGTTGGCCTGCTTCC	615 bp	
**Antibiotic** **Resistance gene**	*blaTEM*	F: CATTTCCGTGTCGCCCTTAT R: TCCATAGTTGCCTGACTCCC	793 bp	[27]

For the PCR analysis, the genomic DNA of isolated *E. faecalis* was extracted using the boiling and chilling method previously described [24]. In brief, 1 mL of the overnight enriched broth culture was centrifuged at 5000 rpm for 5 minutes. After discarding the supernatant, they subjected them to centrifuging again at 5000 rpm for 5 minutes. Then the supernatant was again discarded, and 200 μL of phosphate buffer solution (PBS) was added to the tube, which was then boiled and chilled for 10 minutes. Next, the chilled tubes were centrifuged again for 10 minutes, and the extracted genomic DNA was collected from the supernatant and stored at −20 °C until future use.

A 20 µL of PCR reaction mixture was prepared for amplification, containing 10 µL of master mix (2X) (Promega, Madison, WI, USA), 1 µL of each forward and reverse primer (Table 1)**,** 4 µL of nuclease-free water, and 4 µL (around 50–60 ng) of DNA template. For negative controls in PCR, Non-template controls were included, in which the extracted genomic DNA template was replaced with phosphate-buffered saline (PBS).

After amplification, the PCR products were electrophoresed on a 1.5% agarose gel pre-stained with ethidium bromide. The gel was then visualizedtransilluminator UV using a (Biometra, Göttingen, Germany), with a 100 bp DNA ladder (Promega, Madison, WI, USA).

### Determination of Biofilm-Forming Abilities of *E. faecalis*

To examine the phenotypic evidence of biofilm-forming capability of *E. faecalis,* we used a previously established Congo Red (CR) agar plate test by Randall et al. 2004 [28]. In this qualitative method, Congo red dye binds to extracellular biofilm matrix components, especially amyloid fibers in biofilm-producing bacteria, forming black or dark colonies, whereas non-biofilm-producing bacteria remain red or pink. In brief, *E. faecalis* overnight cultures were streaked onto CRA plates and incubated at 37 °C for 24 h. The strength of biofilm formation was assessed by the apparent changes observed on the CRA plates. Isolates were categorized as strong, intermediate, weak, or non-biofilm formers based on colony appearance: dry, filamentous, crusty black; pink with a dark center; and smooth pink, respectively [29].

### Detection of antibiotic resistance and virulence genes

A simplex PCR assay was used to identify virulence-associated genes, commonly present in *E. faecalis*, including *agg, ace, gelE, fsrB*, and *pil*, which are responsible for aggregation, adhesion, pilus formation, and function as quorum-sensing regulators (Table 1). A 20 µL PCR mixture was prepared, and negative controls were set up by replacing the DNA template with PBS. For positive controls, reactions were used with previously confirmed target DNA to verify the amplification conditions.

### Antibiotic susceptibility testing

The disk diffusion method was used to assess antibiotic susceptibility patterns of E. faecalis isolates in accordance with the Clinical and Laboratory Standards Institute (2024) guidelines [30]. In this study, we selected nine widely available, commonly used antibiotics (HiMedia, Mumbai, Maharashtra, India) as first-line treatment options for many infections in Bangladesh, spanning eight different classes. All the antibiotics represents the three antibiotic categories classified by the World Health Organization (WHO): access group- first line, commonly used (penicillins (ampicillin: 10 μg), tetracyclines (tetracycline: 30 μg), and amphenicols (chloramphenicol: 30 μg), watch group- higher resistance potential (glycopeptides (vancomycin: 30 μg), macrolides (erythromycin: 15 μg), fluoroquinolones (ciprofloxacin: 5 μg, levofloxacin: 5 μg), and ansamycins (rifampin:5 μg), and reserve- last resort antibiotic (oxazolidinones (linezolid: 30 μg) group. Isolates showing resistance to at least three antimicrobial groups were classified as multidrug-resistant (MDR) [31]. The following formula was used to calculate the multiple antibiotic resistance (MAR) [32].


$$\begin{array}{c} MAR\;index=\frac{\text{The quantity of antibiotics to which a specific isolate shows resistance}}{\text{The total quantity of antibiotics used during this investigation}} \end{array}$$

In addition, the *bla*_TEM_ resistance gene was detected by simplex PCR (Table 1).

### Statistical analysis

Excel 365 (Microsoft/Office 365, Redmond, WA, USA) was used to record the data obtained in this study initially, and for further analysis, Statistical Package for Social Sciences (SPSS.v.25, IBM, Chicago, IL, USA) and GraphPad Prism (Prism.v.8.4.2, San Diego, CA, USA) were used. A previously established method described by [33] was used to evaluate the prevalence of various factors associated with E. faecalis isolates by calculating a binomial 95% confidence interval (CI_95_). Any relationship between different degrees of biofilm formation in *E. faecalis* isolates and the presence of virulence genes and phenotypic antibiotic resistance was assessed using the chi-square test in SPSS. Additionally, to examine the potential association between two virulence genes and phenotypic antibiotic resistance in the *E. faecalis* isolates, a bivariate analysis was performed in SPSS. The Pearson correlation coefficient analysis and the chi-square test for relatedness between two variables were performed, with a p-value ≤ 0.05 as the level of significance.

## Results

### Prevalence of *E. faecalis*

**Fig 1:**
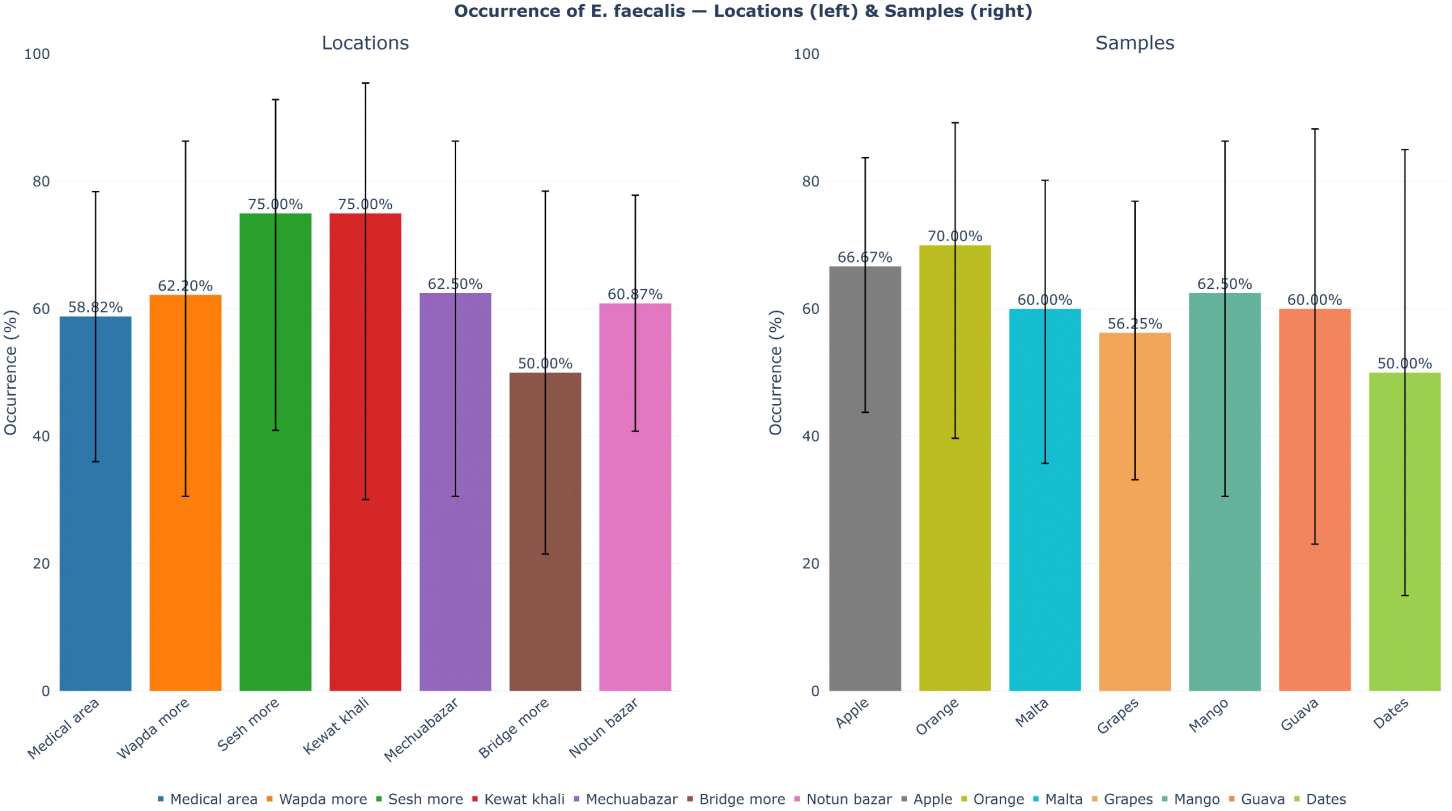
Occurrence (%) of *Enterococcus faecalis* across sampling locations (left panel) and fruit types (right panel). The bar charts represent the percentage of *E. faecalis*-positive samples collected from different market locations (Medical area, Wapda more, Sesh more, Kewat khali, Mechuabazar, Bridge more, and Notun bazar) and fruit types (apple, orange, malta, grapes, mango, guava, and dates). Error bars represent 95% confidence intervals (CI) of the estimated occurrence percentages.

Out of 76 samples, 47 (61.84%) samples were found positive in PCR. The Sesh more and Kewatkhali sites had the highest percentage of PCR-positive isolates, with 75% (6/8) and 75% (3/4), respectively, followed by Mechua bazar, which had 62.5% (5/8), Wapda more 62.2% (5/8), Notun bazar 60.87% (14/23), Medical area 58.82% (10/17), and Bridge more 50% (4/8) (Fig 1**).** A statistically significant difference was found among the locations (p-value = 0.036 < 0.05)**.**

In addition, among the seven types, Orange (70%, 7/10) showed higher PCR positive results than the other six samples types with Apple (66.67%, 12/18), Mango (62.5%, 5/8), Malta (60%, 9/15), Guava (60%, 3/5), Grapes (56.25%, 9/16) and Dates (50%, 2/4). No statistically significant differences were found across sample types (p-value = 0.988 > 0.05) (Fig 1).

### Frequency of biofilm formation

Out of all PCR-positive *E. faecalis*, 18 random samples were tested in Congo red agar to detect biofilm formation ability. 38.88% (7/18) isolates showed strong biofilm-forming ability, 33.33% (6/18) showed intermediate, and 27.78% (5/18) isolates were found to be non-biofilm producers. Furthermore, the frequencies of strong, moderate, and non-biofilm-forming *E. faecalis* isolates showed a statistically nonsignificant variation (Table 2)**.**

**Table 2:** Occurrence of biofilm-forming *E. faecalis* isolates (N = 18).

Name of organism	Nature of Biofilm	Occurrence of biofilm former n (%) ^s^	95% CI (%)	*P value*
*E. faecalis*	Strong	7 (38.88^a^)	20.31 - 61.38	0.779
	Intermediate	6 (33.33^a^)	16.28 - 56.25	
	Weak	5 (27.78^a^)	12.50 - 50.87	

Here, within the variable being evaluated, s = values with different superscripts differ significantly (p < 0.05); N = number of isolates sampled by category; n = number of isolates for the category; CI = confidence interval.

### Virulence genes detection

**Fig 2:**
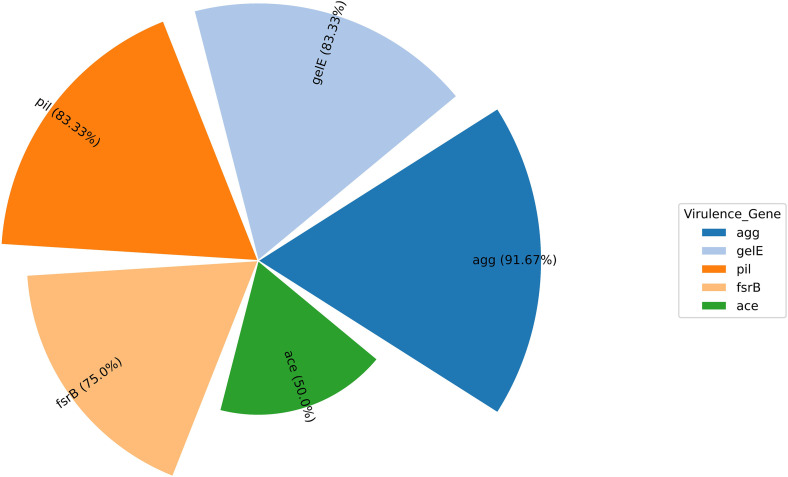
Overall prevalence of virulence genes in 12 isolated *E. faecalis.* The pie chart shows the proportion (%) of isolates positive for each virulence gene (*agg, gelE, pil, fsrB*, and *ace*) detected by PCR. Values represent the percentage of isolates carrying each gene.

Five virulence genes were molecularly detected from randomly chosen 12 PCR-positive isolates. Each isolate contained many virulent genes; *agg* was found in 91.67% (11/12) isolates, followed by *pil* and *gelE,* both at 83.33% (10/12), *fsrB* at 75% (9/12), and *ace* at 50% (6/12) (Fig 2 and S1 Fig).

In bivariate analysis, a statistically significant strong correlation between the *gelE* and *fsrB* gene (Spearman correlation coefficient, ρ = 0.775; p < 0.01). Additionally, a correlation was observed between the *fsrB* and *ace* genes (ρ = 0.577; p < 0.05) (S1 Table).

Regarding biofilm production, strong biofilm former isolates showed higher levels of *agg,* and *pil* genes (85.72%), whereas intermediate biofilm former isolates showed higher levels of *agg* (66.7%), *pil* (50%)*, fsrB* (50%), *gelE* (50%), and *ace* (33.33%)**.**

### Antibiotic resistance profiles of *E. faecalis*

**Fig 3:**
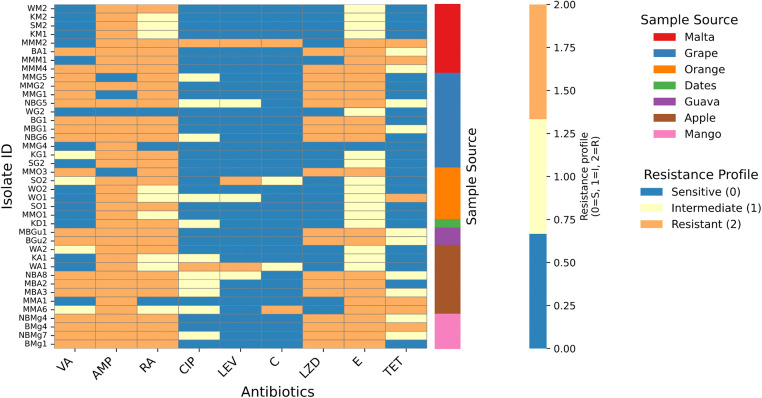
Antibiotic resistance patterns of *E. faecalis* isolates. AMP = ampicillin; C = chloramphenicol; LZD = linezolid; CIP = ciprofloxacin; E = erythromycin; RA = rifampicin; TE = tetracycline; LEV = levofloxacin; VA = vancomycin. Each row represents an individual isolate, and each column represents an antibiotic tested. Susceptibility status is indicated by color: blue = sensitive (0), light yellow = intermediate (1), and orange = resistant (2).

Antibiogram analysis of *E. faecalis* isolates (N = 40) revealed that, within the WHO Access antibiotic group, 90% isolates were phenotypically resistant to ampicillin, 57.5% were resistant to erythromycin, and 15% were resistant to tetracycline, Within the Watch group, 70% were resistant to rifampin, 7.5% were resistant to levofloxacin, 5% were resistant to ciprofloxacin, and 47.5% were resistant to vancomycin. From the Reserve group of antibiotics, 47.5% were resistant to linezolid (Fig 3 and S2 Table). Moreover, 65% of ampicillin-resistant isolates (13/20) contained the *blaTEM* resistance gene. (S2 Fig)

Bivariate statistical analysis showed that the antibiotic-resistant isolates in this study were highly significantly associated with one another. Significant and positive correlation existed between resistance patterns of Vancomycin and Rifampicin (Pearson correlation coefficient, ρ = 0.623; p < 0.01); levofloxacin and ciprofloxacin (ρ = 0.806; p < 0.01), chloramphenicol and ciprofloxacin (ρ = 0.474; p < 0.01), chloramphenicol and levofloxacin (ρ = 0.370; p < 0.05), linezolid and vancomycin (ρ = 1.000; p < 0.01), linezolid and rifampicin (ρ = 0.623; p < 0.01), erythromycin and vancomycin (ρ = 0.818; p < 0.01), erythromycin and rifampicin (ρ = 0.541; p < 0.01), erythromycin and linezolid (ρ = 0.818; p < 0.01), tetracycline and chloramphenicol (ρ = 0.546; p < 0.01) (S3 Table). The correlation analysis should be interpreted with caution, as some strong associations (e.g., linezolid-vancomycin, ρ = 1.000) could be due to the relatively small sample size.

Moreover, Strong biofilm-forming *E. faecalis* isolates exhibited higher resistance to certain antibiotics than intermediate and non-biofilm-forming isolates, particularly to tetracycline (42.9% vs. 0% vs. 20%) and erythromycin (57.1% vs. 33.3% vs. 100%). Ampicillin resistance was uniformly high across all biofilm categories (88.9%), whereas ciprofloxacin, levofloxacin, and chloramphenicol showed the lowest resistance rates (11.1%). Interestingly, vancomycin and linezolid resistance were more prevalent in non-biofilm formers (80%) than in strong (14.3%) or intermediate (33.3%) biofilm producers. However, these differences were not statistically significant (p > 0.05)**.**

The present study identified 12 resistance trends among E. faecalis isolates from the surfaces of fresh fruits. Of 40 PCR-positive samples, 24 (60.00%, CI_95_: 44.6–73.65) isolates showed multidrug resistance (MDR), with MAR index between 0.33 and 0.78 (Table 3).

**Table 3:** Occurrence of MDR E. faecalis isolates and multiple-antibiotic resistance (MAR).

No. of pattern	Antibiotic resistance pattern	No. of antibiotics (classes)	No. of isolates	Overall, MDR isolates %	MAR index
1	AMP, RA, LEV	3(3)	1	24/40 (60.00%)	0.33
2	VA, AMP, RA, LNZ, E	5(5)	15		0.56
3	AMP, RA, CIP, LEV, C, E, TE	7(6)	1		0.78
4	AMP, RA, E, TE	4(4)	1		0.45
5	AMP, E, TE	3(3)	1		0.33
6	VA, AMP, RA, LNZ, E, TE	6(6)	1		0.67
7	AMP, C, E, TE	4(4)	3		0.45
8	VA, RA, LNZ, E	4(4)	1		

**Legends,** R = Resistant; I = Intermediate; S = Sensitive; AMP = Ampicillin; C = Chloramphenicol; LZD = Linezolid; CIP = Ciprofloxacin; E = Erythromycin; RA = Rifampicin; TE = Tetracycline; LEV = levofloxacin and VA = Vancomycin.

## Discussion

*Enterococcus faecalis* is predominantly a commensal bacterium- found in humans, animals, and a variety of habitats, yet it possesses the potential to cause opportunistic infections [34]. Other pathogens can acquire resistance genes from *E. faecalis* strains that are multidrug-resistant (MDR) through horizontal gene transmission [35]. Its capacity to infect humans and animals and acquire genes resistant to antibiotics makes this especially concerning [36].

The overall prevalence of *E. faecalis* in this study was 61.84%; the detection rate was higher in the Seshmore and Kewatkhali areas (75%) and was particularly high in oranges (70%), apples (66.67%), and mangos (62.5%). High population density, poor vendor hygiene, using contaminated or unclean water to wash or spray on fruits to keep them fresh, cross-contamination from display surfaces and human touch, and exposure to dust and wastewater may all contribute to the greater prevalence of *E. faecalis* in samples taken from these areas [37–39]. In addition, the relatively small sample size and uneven sampling distribution due to availability may also contribute to differences in *E. faecalis* occurrence.

Depending on the type of fruits and the source of the sample, similar studies have revealed different prevalence rates. For instance, a study conducted in northern Georgia found that *E. faecalis* was highly prevalent in fruits and vegetables, especially in tomatoes (33%) and radishes (91%) [6]. A recent study by Demisie *et* al. reported 50% prevalence in fruit samples, which is considerably lower than that we found in the vegetable (86.5%) and salad (82.6%) samples [40]. However, a study from Thailand reported 76% of vegetable samples from markets were contaminated with *E. faecalis* [41]. Differences in management practices, wash water quality, fruit handling, market and vendor cleanliness, geographic location, ambient conditions, and the regional microbial burden can all affect variations in the prevalence of *E. faecalis* in raw fruits. Such fruits can be a direct cause of infection when eaten raw [42–44]. This emphasizes the necessity of better cleanliness and secure handling procedures in public marketplaces.

In our study, 38.88% of isolates exhibited strong biofilm-forming ability, 33.33% exhibited intermediate biofilm-forming ability, and 27.78% were non-biofilm producers. Strong biofilm-formers exhibited higher resistance to certain antibiotics compared to intermediate and non-biofilm formers. This could be due to the protective biofilm matrix, which limits antibiotic penetration and upregulates resistance mechanisms, which makes biofilm-associated cells more resilient than cells in intermediate or non-biofilm forms.

Pathogenicity and antibiotic resistance in enterococci may be influenced by the biofilm-forming potential of strong isolates [45]. Biofilm formation is a critical survival strategy that enables *E. faecalis* to persist in hostile environments, evade host immune responses, and resist antimicrobial treatments. A previous study found that *E. faecalis* isolated from the food samples had biofilm-forming capabilities, with 13.3% as strong biofilm-forming *E. faecalis* [46]. The capacity of *E. faecalis* isolates from fruit samples to form biofilms ranged from moderate to strong, suggesting that the bacteria can stick to fruit surfaces and possibly resist handling and storage. This tenacity raises the possibility that consumers will become contaminated, particularly if fruits are consumed raw. As biofilm development increases bacterial survival and tolerance to antibiotics and disinfectants, even strains that seem somewhat resistant to them may still pose a risk of infection. The isolates’ differing abilities to build biofilms suggest that certain strains may be particularly adept at surviving on fruits; therefore, proper handling and strict hygiene are essential from farm to table.

The main findings of this study were that *E. faecalis* isolates may frequently have multiple virulence genes. This study detected five different virulence genes in the *E. faecalis* isolated from the surfaces of fresh fruits. All *E. faecalis* isolates from fruit samples were positive for multiple virulence genes, with *agg, ace, gelE, fsrB*, and pil detected in 91.67%, 50%, 83.33%, 75%, and 83.33% of the isolates, respectively*.* In our study, a strong positive correlation between gelE and fsrB was observed, reflecting their functional linkage: *fsrB* is part of the quorum-sensing system that regulates *gelE* expression, leading to their frequent co-occurrence. In contrast, the moderate correlation between *fsrB* and *ace* suggests possible co-selection within the same isolates, although this relationship is less direct and may be influenced by other genetic or environmental factors. Very few studies have investigated the presence of *E. faecalis* on fresh fruit surfaces, and even fewer have characterized their virulence profiles. In one study, 74 potential virulence genes were found in *E. faecalis* isolates from salad samples using whole-genome sequencing, with 17–44 genes per isolate [41]. The presence of the adhesion genes, *ace* (100%) and *efaA* (100%), the biofilm-associated *fsr* operon, gelatinase (*gelE*, 100%), and metalloprotease *sprE* was among the important discoveries (100%). However, a study in Korea found that 38.1% of *gelE* genes from *E. faecalis* were isolated from various food sources, which is far lower than our results [47]. Others reported that, among *E. faecalis* isolates from fish, vegetables, and humans, the most frequent virulence gene was *gelE* (65.38%), followed by *ace* (51.92%), *cylA* (48.08%), *asa1* (40.38%), and *esp* (25%), while *hyl* was the least frequent gene (1.92%) [48]. The genes ace, *fsrC, gelE*, and *sprE*, which are linked to attachment and biofilm formation in *E. faecalis*, showed clear positive relationships [49]. Since their presence on raw fruits represents a potential route for foodborne transmission of pathogenic and multidrug-resistant *E. faecalis*, strict hygiene during handling, purchasing, and processing is necessary.

In this study, high resistance was observed to ampicillin (90%) and rifampicin (70%), while moderate resistance was observed to erythromycin (57.5%), vancomycin (47.5%), and linezolid (47.5%). Lower resistance levels were recorded for tetracycline (15%), levofloxacin (7.5%), ciprofloxacin (5%), and chloramphenicol (5%). Ampicillin is one of the highly prescribed antibiotics in Bangladesh [50,51], which suggests widespread environmental distribution and aligns with our findings. Among the 20 randomly selected Ampicillin-resistant isolates, 65% were positive for *bla*_TEM_, suggesting the presence of mobile resistance determinants that facilitate horizontal gene transfer. In this study, we used the disc diffusion method to test vancomycin against our isolates, which showed 47.5% resistance. Although an MIC-based confirmation was needed, the 47.5% isolates resistant to vancomycin are alarming.

The prevalence of vancomycin resistance (47.5%) on fruit surfaces suggests possible hospital-acquired contamination by enterococci. VRE on fruits indicates that resistance genes can be acquired through environmental exposure or horizontal gene transfer, even in populations not directly exposed to antibiotics. This emphasizes the importance of handling and selling fruit with strict hygiene measures to reduce the risk of human transmission of resistant *E. faecalis*. Studies carried out in a variety of geographical locations, including Ghana [52], Japan [53], the USA [6], and Brazil [54], have revealed that enterococci isolated from different foods exhibit varying degrees of AMR against a range of antibiotics. Strong positive correlations between multiple antibiotic co-resistance patterns were revealed by bivariate analysis of antibiotic-resistant *E. faecalis* from fresh fruits, including vancomycin-rifampicin (ρ = 0.623), levofloxacin-ciprofloxacin (ρ = 0.806), linezolid–vancomycin (ρ = 1.000), erythromycin–vancomycin (ρ = 0.818), and tetracycline–chloramphenicol (ρ = 0.546), indicating that these resistances frequently co-occur within the same isolates, likely due to co- selection or linked resistance mechanisms. It is important to note that the correlation analysis was exploratory in nature and involved multiple pairwise comparisons, which increases the risk of Type I error. No formal correction for multiple testing was applied; therefore, some observed associations may represent false positives. Furthermore, some strong correlations may reflect the limited sample size; therefore, the findings should be interpreted cautiously. 60.00% of isolates were classified as MDR, with MAR index values ranging from 0.33 to 0.78. These MDR isolates exhibited a variety of resistance patterns indicative of either significant selection pressure or exposure to antimicrobials. Despite the growing concern, research on the multidrug resistance (MDR) profiles of *E. faecalis* isolated from fruit surfaces remains scarce. But similar findings were reported in one study, in which 29.8% of bacterial strains isolated from lettuce and fruits were multidrug-resistant (MDR) [55]. Another study found a 67.93% prevalence of MDR in bacterial isolates recovered from fresh fruits and vegetables [56]. The presence of MDR *E. faecalis* on fruits is particularly alarming, as consumption of such contaminated fruits could facilitate the transmission of resistant strains to humans. This not only increases the risk of difficult-to-treat infections but also contributes to the potential dissemination of resistance genes within the community ecosystem.

This study provides novel insights into multidrug-resistant, biofilm-forming *E. faecalis* on fresh fruits in Bangladesh. However, several limitations should be acknowledged. The sampling was restricted to a single city, and biofilm formation was assessed only qualitatively, with partial screening for resistance and virulence genes. Therefore, the observed prevalence and distribution of virulence traits and biofilm-associated characteristics may be over- or under-estimated and may not fully represent all isolates or broader populations. In addition, whole-genome sequencing was not performed, precluding comprehensive analysis of clonal relationships, mobile genetic elements, and the complete resistome. Antimicrobial susceptibility testing was performed using the disc diffusion method in accordance with Clinical and Laboratory Standards Institute guidelines. This approach has recognized limitations for certain antibiotic classes, such as glycopeptides and oxazolidinones, where MIC-based methods are preferred for greater accuracy.

Despite these constraints, the findings provide a region-specific baseline and underscore the need for future studies that incorporate larger-scale sampling, comprehensive molecular profiling, and genomic analysis to better elucidate transmission dynamics and potential public health impacts.

## Conclusion

To the best of our knowledge, this study is the first to report baseline site-specific data on resistance profile, virulence determinants, and biofilm-forming ability of *E. faecalis* isolated from the surface of fresh fruits sold in and around Mymensingh city, Bangladesh. Despite the risk of overestimation due to resource limitations, the findings suggest a concerning presence of multidrug-resistant (MDR) *E. faecalis* capable of forming biofilms and carrying virulence genes, with potential resistance to critically important antibiotics such as vancomycin and linezolid. These results indicate that fresh fruits may serve as carriers of MDR *E. faecalis* and pose a risk of transmission to humans, leading to public health threats. This study highlights the urgent need for strict enforcement of market hygiene practices in fruit handling, storage, and distribution. Furthermore, it underscores the importance of continuous food safety monitoring and routine AMR surveillance to track the dissemination of antimicrobial resistance in fresh produce, particularly fruits, and to protect public health in Bangladesh. The baseline data from this study will help future studies to focus on investigating whole-genome sequence and transmission patterns of MDR *E. faecalis* in a public health context, and on conducting a longitudinal study to identify risk factors that could help prevent the dissemination of AMR and pathogenicity.

## Supporting information

S1 Fig PCR amplification of primers used to detect virulence gene. (a) PCR detection of *agg* gene with amplicon size 413 bp; (b) PCR detection of *gelE* gene with amplicon size 704 bp; (c) PCR detection of *pil* gene with amplicon size 620 bp; (d) PCR detection of *ace* gene with amplicon size 615 bp (Left); and *fsrB,* gene with amplicon size 428 bp (Right). In all cases, L: 100 bp DNA ladder; NC: negative control; PC: positive control).
(DOCX)

S2 Fig PCR amplification of primers used to detect resistance gene, PCR detection of *blaTEM* gene with amplicon size 793 bp; L: 100 bp DNA ladder; NC: negative control; PC: positive control. (DOCX)

S1 Table Correlation between two virulence genes in *E. faecalis* isolated from fruits. (DOCX)

S2 Table Antibiotic resistance profile of *E. faecalis* from different sources of samples. (DOCX)

S3 Table Correlation between two resistant antibiotics in *E. faecalis* isolated from fresh fruit samples. (DOCX)
