# Supplementary material for: Fresh fruit surface: A potential source for multidrug-resistant biofilm-forming Enterococcus faecalis having potential public health significance
Source: PLoS One. 2026 Aug 5;21(8):e0355410. doi: 10.1371/journal.pone.0355410 (PMC13440884; doi:10.1371/journal.pone.0355410)
Supplement: Supplementary file 1 [file pone.0355410.s006.pdf]

PLOS ONE

Fresh fruit surface: a potential source for multidrug-resistant biofilm-forming Enterococcus faecalis having potential public health significance

--Manuscript Draft--

**Manuscript Number:**	PONE-D-25-60195
**Article Type:**	Research Article
**Full Title:**	Fresh fruit surface: a potential source for multidrug-resistant biofilm-forming Enterococcus faecalis having potential public health significance
**Short Title:**	Enterococcus faecalis on Fresh fruit surface
**Corresponding Author:**	Md. Tanvir Rahman, DVM, MSc (Canada), PhD (UK), FBAS<br>Bangladesh Agricultural University<br>Mymensingh, Mymensingh BANGLADESH
**Keywords:**	Enterococcus faecalis; Opportunistic pathogens; fruit surface; antibiotic resistance; biofilm; virulence
**Abstract:**	Fruits are important in the diet because of their nutrients, vitamins, and minerals needed for a person to lead a healthy life. Enterococci are opportunistic pathogens, but may often cause severe infections. Fruit surfaces can act as a potential source for antibiotic-resistant and zoonotic enterococci. This work aimed to isolate Enterococcus faecalis from fruit surfaces, detect its virulence genes and biofilm-forming ability, and determine its antibiotic resistance pattern. A total of 76 samples (18 apples, 10 oranges, 15 maltas, 16 grapes, 8 mangoes, 5 guavas, 4 dates) were collected and surface-washed, and were used to isolate and identify Enterococcus faecalis by cultural and biochemical tests, followed by polymerase chain reaction (PCR). The antibiotic resistance patterns of positive isolates were determined by the disc diffusion method against nine common antibiotics. Out of 76 samples, 47 (61.84%) fruit samples were found to be positive for E. faecalis. In the antibiogram study, the highest resistance was found against ampicillin (90.00%), followed by rifampicin (70.00%), erythromycin (57.50%), vancomycin, and linezolid (47.50%). The ampicillin-resistance gene blaTEM was present in 65% of the isolates. 60% isolates showed multidrug resistance (MDR), with the MAR index also fluctuating between 0.33 and 0.78. The isolated enterococci were also subjected to biofilm assay using the Congo red agar test, and found that 38.88% of the isolates from fruit surfaces showed strong biofilm-forming ability, 33.33% showed intermediate, and 27.78% of isolates showed no sign of producing biofilm. In PCR, virulence genes agg (91.67%), ace (50%), gelE (83.33%), fsrB (75%), and pil (83.33%) genes were detected. These findings suggest that fruit surfaces harbor antibiotic-resistant E. faecalis, which should be monitored through One Health strategies to reduce the potential risk for human health.
**Order of Authors:**	Rownak Jahan
	Naeem Ahammed Ibrahim Fahim
	Md. Abdullah Evna Hasan
	Md. Liton Rana
	Md. Tabeer Hossain Antor
	Samia Salam
	Rony Ibne Masud
	Dilruba Akter Jany
	Zuhayr Bakhtiyar
	Md. Saiful Islam
	Muhammad Tofazzal Hossain
	Md. Tanvir Rahman, DVM, MSc (Canada), PhD (UK), FBAS
**Additional Information:**	

Powered by Editorial Manager® and ProduXion Manager® from Aries Systems Corporation

Question	Response
**Financial Disclosure**<br><br>Enter a financial disclosure statement that describes the sources of funding for the work included in this submission. Review the submission guidelines for detailed requirements. View published research articles from *PLOS ONE* for specific examples.<br><br>This statement is required for submission and **will appear in the published article** if the submission is accepted. Please make sure it is accurate.<br><br>**Funded studies**<br>Enter a statement with the following details:<br>• Initials of the authors who received each award<br>• Grant numbers awarded to each author<br>• The full name of each funder<br>• URL of each funder website<br>• Did the sponsors or funders play any role in the study design, data collection and analysis, decision to publish, or preparation of the manuscript?<br><br>Did you receive funding for this work?	Yes
Please add funding details.<br>as follow-up to "**Financial Disclosure**<br><br>Enter a financial disclosure statement that describes the sources of funding for the work included in this submission. Review the submission guidelines for detailed requirements. View published research articles from *PLOS ONE* for specific examples.<br><br>This statement is required for submission and **will appear in the published article** if the submission is accepted. Please make sure it is accurate.	The authors extend their appreciation to the Bangladesh Agricultural University Research System (BAURES) (grant No. 2024/141/BAU) for partial funding of the research work. The funders had no role in the study design, data collection, and analysis, decision to publish, or preparation of the manuscript.

**Funded studies**<br>Enter a statement with the following details:<br>• Initials of the authors who received each award<br>• Grant numbers awarded to each author<br>• The full name of each funder<br>• URL of each funder website<br>• Did the sponsors or funders play any role in the study design, data collection and analysis, decision to publish, or preparation of the manuscript?<br><br>Did you receive funding for this work?"	
Please select the country of your main research funder (please select carefully as in some cases this is used in fee calculation).<br>as follow-up to "**Financial Disclosure**<br><br>Enter a financial disclosure statement that describes the sources of funding for the work included in this submission. Review the submission guidelines for detailed requirements. View published research articles from *PLOS ONE* for specific examples.<br><br>This statement is required for submission and **will appear in the published article** if the submission is accepted. Please make sure it is accurate.<br><br>**Funded studies**<br>Enter a statement with the following details:<br>• Initials of the authors who received each award<br>• Grant numbers awarded to each author<br>• The full name of each funder<br>• URL of each funder website<br>• Did the sponsors or funders play any role in the study design, data collection and analysis, decision to publish, or preparation of the manuscript?<br><br>Did you receive funding for this work?"	BANGLADESH - BD
**Competing Interests**	The authors have declared that no competing interests exist.

Use the instructions below to enter a competing interest statement for this submission. On behalf of all authors, disclose any competing interests that could be perceived to bias this work—acknowledging all financial support and any other relevant financial or non-financial competing interests.<br><br>This statement is required for submission and **will appear in the published article** if the submission is accepted. Please make sure it is accurate and that any funding sources listed in your Funding Information later in the submission form are also declared in your Financial Disclosure statement.<br><br>View published research articles from *PLOS ONE* for specific examples.<br><br>**NO authors have competing interests**<br><br>Enter: *The authors have declared that no competing interests exist.*<br><br>**Authors with competing interests**<br><br>Enter competing interest details beginning with this statement:<br><br>*I have read the journal's policy and the authors of this manuscript have the following competing interests: [insert competing interests here]*<br><br>* typeset	
**Ethics Statement**<br><br>Enter an ethics statement for this submission. This statement is required if the study involved:<br><br>• Human participants<br>• Human specimens or tissue<br>• Vertebrate animals or cephalopods<br>• Vertebrate embryos or tissues	N/A

• Field research<br><br>Write "N/A" if the submission does not require an ethics statement.<br><br>General guidance is provided below. Consult the submission guidelines for detailed instructions. **Make sure that all information entered here is included in the Methods section of the manuscript.**	

Powered by Editorial Manager® and ProduXion Manager® from Aries Systems Corporation

**Format for specific study types**<br><br>**Human Subject Research (involving human participants and/or tissue)**<br>• Give the name of the institutional review board or ethics committee that approved the study<br>• Include the approval number and/or a statement indicating approval of this research<br>• Indicate the form of consent obtained (written/oral) or the reason that consent was not obtained (e.g. the data were analyzed anonymously)<br><br>**Animal Research (involving vertebrate animals, embryos or tissues)**<br>• Provide the name of the Institutional Animal Care and Use Committee (IACUC) or other relevant ethics board that reviewed the study protocol, and indicate whether they approved this research or granted a formal waiver of ethical approval<br>• Include an approval number if one was obtained<br>• If the study involved *non-human primates*, add *additional details* about animal welfare and steps taken to ameliorate suffering<br>• If anesthesia, euthanasia, or any kind of animal sacrifice is part of the study, include briefly which substances and/or methods were applied<br><br>**Field Research**<br><br>Include the following details if this study involves the collection of plant, animal, or other materials from a natural setting:<br>• Field permit number<br>• Name of the institution or relevant body that granted permission	
**Data Availability**<br><br>Authors are required to make all data underlying the findings described fully available, without restriction, and from the time of publication. PLOS allows rare exceptions to address legal and ethical concerns. See the PLOS Data Policy and FAQ for detailed information.	Yes - all data are fully available without restriction

A Data Availability Statement describing where the data can be found is required at submission. Your answers to this question constitute the Data Availability Statement and **will be published in the article**, if accepted.<br><br>**Important:** Stating 'data available on request from the author' is not sufficient. If your data are only available upon request, select 'No' for the first question and explain your exceptional situation in the text box.<br><br>Do the authors confirm that all data underlying the findings described in their manuscript are fully available without restriction?	
**Describe where the data may be found in full sentences. If you are copying our sample text, replace any instances of XXX with the appropriate details.**<br><br>• If the data are **held or will be held in a public repository**, include URLs, accession numbers or DOIs. If this information will only be available after acceptance, indicate this by ticking the box below. For example: *All XXX files are available from the XXX database (accession number(s) XXX, XXX.).*<br>• If the data are all contained **within the manuscript and/or Supporting Information files**, enter the following: *All relevant data are within the manuscript and its Supporting Information files.*<br>• If neither of these applies but you are able to provide **details of access elsewhere**, with or without limitations, please do so. For example:<br><br>*Data cannot be shared publicly because of [XXX]. Data are available from the XXX Institutional Data Access / Ethics Committee (contact via XXX) for researchers who meet the criteria for access to confidential data.*<br><br>*The data underlying the results presented in the study are available from (include the name of the third party*	All relevant data are within the manuscript and its Supporting Information files.

and contact information or URL).<br>• This text is appropriate if the data are owned by a third party and authors do not have permission to share the data.<br><br>* typeset	
Additional data availability information:	Tick here if the URLs/accession numbers/DOIs will be available only after acceptance of the manuscript for publication so that we can ensure their inclusion before publication.

Powered by Editorial Manager® and ProduXion Manager® from Aries Systems Corporation

Click here to access/download;Manuscript;Manuscript.docx

Fresh fruit surface: a potential source for multidrug-resistant biofilm-forming Enterococcus faecalis having potential public health significance

Rownak Jahan[1], Naeem Ahammed Ibrahim Fahim[1], Md. Abdullah Evna Hasan[1], Md. Liton Rana[1, 2, 3], Md. Tabeer Hossain Antor[1], Samia Salam[1], Rony Ibne Masud[1], Dilruba Akter Jany[1], Zuhayr Bakhtiyar[1], Md. Saiful Islam[1,4], Muhammad Tofazzal Hossain[1], and Md. Tanvir Rahman[1]*

[1]Department of Microbiology and Hygiene, Faculty of Veterinary Science, Bangladesh Agricultural University, Mymensingh 2202, Bangladesh.

[2]National Engineering Research Center of Industrial Wastewater Detoxication and Resource Recovery, Research Center for Eco-Environmental Sciences, Chinese Academy of Sciences, Beijing 100085, China

[3]University of Chinese Academy of Sciences, Beijing 100049, China

[4]Department of Animal Science, University of California - Davis, Davis 95616, United States

*Corresponding author

E-mail: tanvirahman@bau.edu.bd

// Abstract

Fruits are important in the diet because of their nutrients, vitamins, and minerals needed for a person to lead a healthy life. Enterococci are opportunistic pathogens, but may often cause severe infections. Fruit surfaces can act as a potential source for antibiotic-resistant and zoonotic enterococci. This work aimed to isolate *Enterococcus faecalis* from fruit surfaces, detect its virulence genes and biofilm-forming ability, and determine its antibiotic resistance pattern. A total of 76 samples (18 apples, 10 oranges, 15 maltas, 16 grapes, 8 mangoes, 5 guavas, 4 dates) were collected and surface-washed, and were used to isolate and identify Enterococcus faecalis by cultural and biochemical tests, followed by polymerase chain reaction (PCR). The antibiotic resistance patterns of positive isolates were determined by the disc diffusion method against nine common antibiotics. Out of 76 samples, 47 (61.84%) fruit samples were found to be positive for *E. faecalis*. In the antibiogram study, the highest resistance was found against ampicillin (90.00%), followed by rifampicin (70.00%), erythromycin (57.50%), vancomycin, and linezolid (47.50%). The ampicillin-resistance gene *blaTEM* was present in 65% of the isolates. 60% isolates showed multidrug resistance (MDR), with the MAR index also fluctuating between 0.33 and 0.78. The isolated enterococci were also subjected to biofilm assay using the Congo red agar test, and found that 38.88% of the isolates from fruit surfaces showed strong biofilm-forming ability, 33.33% showed intermediate, and 27.78% of isolates showed no sign of producing biofilm. In PCR, virulence genes *agg* (91.67%), *ace* (50%), *gelE* (83.33%), *fsrB* (75%), and *pil* (83.33%) genes were detected. These findings suggest that fruit surfaces harbor antibiotic-resistant *E. faecalis*, which should be monitored through One Health strategies to reduce the potential risk for human health.

Keywords: *Enterococcus faecalis*, Opportunistic pathogens, fruit surface, antibiotic resistance, biofilm, virulence.

Introduction

Enterococci are commensal bacteria found in the intestines of domestic animals and humans. They may also be found in the environment, including soil, water, plants, insects, birds, and wild animals. Currently, they rank third among the organisms often found in nosocomial infections [1]. *Enterococcus faecalis* and *Enterococcus faecium* can cause bacteremia, infective endocarditis, urinary tract infections, and wound infections in people [2]. Enterococci can also infect the central nervous system [3]. 80% to 90% of human enterococcal infections are caused by *E. faecalis*, with the bulk of the remaining infections being caused by *E. faecium*. Among the various types of antibiotic-resistant enterococci, vancomycin-resistant enterococci (VRE) are a major global public health concern [4].

A healthy diet should include fruits, as they are rich in vitamins, minerals, fiber, and antioxidants, which are essential for maintaining good health, supporting immune function, and aiding digestion. Considering the nutritional aspects of fruits, fruit consumption is increasing in a significant way day by day. However, fruit food safety is most seriously threatened by microbial contamination, particularly foodborne pathogens, as large and varied bacterial communities may be found in fresh fruits and vegetables [5]. Sometimes, fresh fruits can be a source of entry point if they are eaten without washing or proper cleaning, making pathogen contamination a major consumer concern [6,7]. Climate change, excessive pesticide usage, inadequate postharvest handling and cleanliness, and fruit contamination all raise the danger of foodborne illnesses, financial losses, and food insecurity [8]. Enteropathogenic bacteria on fruits that are skin-edible also increase consumer health risks. More than ten pathogenic organisms were isolated from fresh-cut fruits in China, highlighting the potential role of fruits in spreading the microorganisms among community

members [9]. Additionally, linked to food-borne infections, enterococci can be polluted when store vendors use tainted water for cleaning and washing [10].

The development and spread of antibiotic resistance in bacteria are well-known global health concerns. Enterococci could be reservoirs of antibiotic resistance and are becoming increasingly alarming [6,11]. Consuming fresh fruit can introduce bacteria resistant to antibiotics, which can raise the body's baseline level of antibiotic resistance and result in infections that are hard or impossible to cure with existing drugs [12]. Additionally, raw foods have been found to have antibiotic-resistant *E. faecalis*, which may be connected to irrigation water that is directly passed to people [13]. The presence of multidrug-resistant (MDR) genes in pathogenic bacteria associated with fresh fruits and liquids also raises safety concerns. The lack of antibiotic targets, low-affinity targets, impermeability to some antibiotics, efflux pumps, and lack of uptake pathways for different antimicrobials are examples of E. faecalis's inherent resistance mechanisms [14,15]. The lack of antibiotic targets, low-affinity targets, impermeability to some antibiotics, efflux pumps, and lack of uptake pathways for different antimicrobials are examples of E. faecalis's inherent resistance mechanisms [16]. Due to the unique combination of intrinsic and acquired antibiotic tolerance, Esp and Ebp proteins enable *E. faecalis* to form modestly organized but extremely persistent biofilms, making its infections particularly challenging to treat [17]. It is still unknown how E. faecalis persists and adapts to different environments, despite recent research identifying new resistance elements, gene transfer events, and surface proteins. Meta-analyses have revealed that virulence genes (*esp*, *gelE*) and resistance genes (*vanA*, *ermB*) co-occur in clinical and environmental isolates, suggesting horizontal gene transfer [18–20].

To the best of our knowledge, no prior study has been conducted on antibiotic resistance, biofilm, and virulence factors of *E. faecalis* isolated from fresh fruit surfaces in Bangladesh, especially

around the Mymensingh region. In this study, we aimed to address these knowledge gaps and examine how fresh fruits may contribute to transmitting *E. faecalis* and spreading antibiotic resistance.

Materials and Methods

Sample Collection and Preparation

Between May 2024 and September 2024, 76 samples from various fruit shops, located in Chorpara (24.7464°N, 90.4092°E), Sesh more (24.7178°N, 90.4442°E), Kewatkhali (24.7316°N, 90.4163°E), Wapda more(24.7460°N, 90.4179°E), Bridge more (24.7470°N, 90.4200°E), Mechua Bazar (24.7558°N, 90.4100°E), and Notun Bazar (24.7589°N, 90.4025°E) were collected around Mymensingh town (24.7460°N, 90.4179°E) in Bangladesh. These samples included 18 apples (*Malus domestica*), 10 oranges (*Citrus sinensis*),15 maltas (*Citrus reticulata*), 16 grapes (*Vitis vinifera*), 8 mangoes (*Mangifera indica*), 5 guavas (*Psidium guajava*), and 4 dates (*Phoenix dactylifera*). The areas were chosen for the study, located close to the medical entrances and in various locations throughout the town, including Bangladesh Agricultural University. Maintaining a cold chain, all the fruits were moved to a separate zip-lock bag with a unique tag number and sent directly to the Department of Microbiology and Hygiene's Bacteriology laboratory (24.7245° N, 90.4372° E). Each sample was washed with 10 ml PBS, and from each sample, 100 µl of washed PBS was transferred to a sterile Eppendorf tube containing 1 ml of nutrient broth (HiMedia, India). The test tubes were then incubated aerobically overnight at 37°C to promote bacterial growth [21,22].

Isolation and Molecular Identification of *E. faecalis*

The primary isolation of *E. faecalis* was done by culture method on an Enterococcus Agar Base (EAB) (HiMedia, Mumbai, Maharashtra, India) medium. A loopful of inoculate from the enriched

culture was streaked onto EAB Petri dish and incubated at 37°C for 16 to 24 hours. A yellowish circular colony on EAB medium was considered as *E. faecalis* and further confirmed by Gram's staining and multiple biochemical tests, including sugar fermentation test, Voges–Proskauer test, indole test, and catalase activity. Finally, *E. faecalis* was identified at the molecular level by targeting $ddl_{E.\ faecalis}$ gene using simplex PCR as described by [23]. **(Table 1).**

For the PCR analysis, the genomic DNA of isolated *E. faecalis* was extracted using the boiling and chilling method previously described earlier [24]. In brief, 1 mL of the overnight enriched broth culture was centrifuged at 5000 rpm for 5 minutes. After discarding the supernatant, they subjected them to centrifuging again at 5000 rpm for 5 minutes. Then the supernatant was again discarded, and 200 µL of phosphate buffer solution (PBS) was added to the tube and boiled and chilled for 10 minutes, respectively. Next, the chilled tubes were centrifuged again for 10 minutes, and the extracted genomic DNA was collected from the supernatant and stored at -20 °C until future use.

A 20 µL of PCR reaction mixture was prepared for amplification, containing 10 µL of master mix (2X) (Promega, Madison, WI, USA), 1 µL of each forward and reverse primer **(Table 1),** 4 µL of nuclease-free water, and 4 µL of DNA template. For negative controls in PCR, Non-template controls were included in which the extracted genomic DNA template was replaced with phosphate buffer solution (PBS).

After the completion of the amplification step, the PCR products underwent electrophoresis on a 1.5% agarose gel pre-stained with ethidium bromide. The gel was then visualized using a UV transilluminator (Biometra, Göttingen, Germany), with a 100 bp DNA ladder (Promega, Madison, WI, USA).

Table 1. List of primers used for specific detection of *E. faecalis* isolates with their virulence and antibiotic resistance genes

Target Organism	Target genes	Primer sequence (5′-3′)	Amplicon size	References
E. faecalis	*ddl*	F: ATCAAGTACAGTTAGTCTT<br>R: ACGATTCAAAGCTAACTG	941 bp	[25]
**Virulence gene**	*gelE*	F: GGTGAAGAAGTTACTCTGAC<br>R: GGTATTGAGTTATG*AGG*GGC	704 bp	[26]
	agg	F: TCTTGGACACGACCCATGAT<br>R: AGAAAGAACATCACCACGAC	413 bp	
	pil	F: GAAGAAACCAAAGCACCTAC<br>R: CTACCTAAGAAAAGAAACGG	620 bp	
	fsrB	F: TAATCT*AGG*CTTAGTTCCCAC<br>R: CTAAATGGCTCTGTCGTCTAG	428 bp	
	ace	F: GAATGACCGAGAACGATGGC<br>R: CTTGATGTTGGCCTGCTTCC	615 bp	
**Antibiotic Resistance gene**	*blaTEM*	F: CATTTCCGTGTCGCCCTTAT<br>R: TCCATAGTTGCCTGACTCCC	793 bp	[27]

Determination of Biofilm-Forming Abilities of *E. faecalis*

To examine the phenotypic evidence of biofilm formation capability of *E. faecalis,* we considered a previously established Congo Red (CR) agar plate test by [28].To detect the biofilm formation, at first, *E. faecalis* overnight cultures were streaked on the CRA plates and then incubated at 37 °C for 24 h. The strength of biofilm formation was detected based on the apparent changes in the CRA plates. Isolates were categorized as strong, intermediate, weak, or non-biofilm formers according to their colony appearances: dry, filamentous, crusty black; pink with a dark center; and smooth pink, respectively [29].

Detection of Virulence Genes

A simplex PCR assay was done to identify virulence-associated genes commonly present in *E. faecalis*, including *agg*, *ace*, *gelE*, *fsrB*, and *pil* genes **(Table 1)**. A 20µL PCR mixture was prepared, and negative controls were set up by replacing the DNA template with PBS. Whereas, for positive controls, reactions consisted of previously confirmed target DNA to verify the amplification conditions.

Antibiotic Susceptibility Testing

The disk diffusion method was employed to assess the antibiotic susceptibility patterns of *E. faecalis* isolates by following the guidelines of the Clinical and Laboratory Standards Institute (2024) [30]. In this study, nine antibiotics (HiMedia, Mumbai, Maharashtra, India), which represent eight different classes of antibiotics, were used. All the antibiotics represents the three antibiotic categories classified by the World Health Organization (WHO): access (penicillins (ampicillin: 10 µg), tetracyclines (tetracycline: 30 µg), and amphenicols (chloramphenicol: 30 µg) group, watch (glycopeptides (vancomycin: 30 µg), macrolides (erythromycin: 15 µg), fluoroquinolones (ciprofloxacin: 5 µg, levofloxacin: 5 µg), and ansamycins (rifampin:5 µg) group, and reserve (oxazolidinones (linezolid: 30 µg) group. Isolates showing resistance to at least three antimicrobial groups were classified as multidrug-resistant (MDR) [31]. The following formula was used to calculate the multiple antibiotic resistance (MAR) [32].

$$\text{MAR index} = \frac{\text{The quantity of antibiotics to which a specific isolate shows resistance}}{\text{The total quantity of antibiotics used during this investigation}}$$

In addition, resistance gene *bla*TEM was also detected using a simplex PCR method **(Table 1).**

Statistical Analysis

Excel 365 (Microsoft/Office 365, Redmond, WA, USA) was used to record the data obtained in this study initially, and for further analysis, Statistical Package for Social Sciences (SPSS.v.25,

IBM, Chicago, IL, USA) and GraphPad Prism (Prism.v.8.4.2, San Diego, CA, USA) was used. A previously established method described by [33]was followed to evaluate the prevalence of various factors related to the *E. faecalis* isolates by calculating a binominal 95% confidence interval (CI_{95}). Any relationship between different degrees of biofilm formation in *E. faecalis* isolates and the presence of virulence genes and phenotypic antibiotic resistance were assessed using chi-square test in SPSS. Additionally, to examine the potential association between two virulence genes and phenotypic antibiotic resistance in the *E. faecalis* isolates, a bivariate analysis was performed in SPSS. A *p*-value of less than 0.05 ($p < 0.05$) indicated statistical significance.

Results

Prevalence of *E. faecalis*

Out of 76 samples, 47 (61.84%, CI_{95}: 50.60–71.94) samples were found positive in PCR. The Sesh more and Kewatkhali sites had the highest percentage of PCR-positive isolates, with 75% (6/8, CI_{95}: 40.93–92.85) and 75% (3/4, CI_{95}: 30.06–95.44), respectively, followed by Mechua bazar, which had 62.5% (5/8, CI_{95}: 30.57–86.32), Wapda more 62.2% (5/8, CI_{95}: 30.57–86.32), Notun bazar 60.87% (14/23, CI_{95}: 50.60–71.94), Medical area 58.82% (10/17, CI_{95}: 36.01–78.39) and Bridge more 50% (4/8, CI_{95}: 21.52–78.48) (**Fig 1).**

In addition, among the seven types, Orange (70%, CI_{95}: 39.68–89.22) showed higher PCR positive results than the other six samples types with Apple (66.67%, CI_{95}: 43.75–83.72), Mango (62.5%, CI_{95}: 30.57–86.32), Malta (60%, CI_{95}: 35.75–80.18), Guava (60%, CI_{95}: 23.07–88.24), Grapes (56.25%, CI_{95}: 33.18–76.90) and Dates (50%, CI_{95}: 15.00–85.00). No statistically significant differences were found across various sample types (p-value > 0.05) (**Figure 1).**

Fig 1: Occurrence of *E. faecalis* in different samples and locations

Frequency of Biofilm Formation

Out of all PCR-positive *E. faecalis*, 18 random samples were tested in Congo red agar to detect biofilm formation ability. 38.88% (7/18, CI_{95}: 20.31 - 61.38) isolates showed strong biofilm-forming ability, 33.33% (6/18, CI_{95}: 16.28 - 56.25) showed intermediate, and 27.78% (5/18, CI_{95}: 12.50 - 50.87) isolates were found to be non-biofilm producers. Furthermore, the frequencies of strong, moderate, and non-biofilm-forming *E. faecalis* isolates showed a statistically nonsignificant variation **(Table 2).**

Table 2. Occurrence of biofilm-forming *E. faecalis* isolates (N = 18)

**Name of organism**	**Nature of Biofilm**	**Occurrence of biofilm formers n (%)** ^{s}	**95% CI (%)**	***P value***
E. faecalis	Strong	7 (38.88^{a})	20.31 - 61.38	0.779
	Intermediate	6 (33.33^{a})	16.28 - 56.25	
	Weak	5 (27.78^{a})	12.50 - 50.87	

Here, within the variable being evaluated, s = values with different superscripts differ significantly ($p < 0.05$), N = number of isolates sampled by category, n = no of isolates for the category, CI = confidence interval.

Virulence Genes Detection

Five virulence genes were molecularly detected from randomly chosen 12 PCR-positive isolates. Each isolate contained many virulent genes, *agg* was found in 91.67% (11/12, CI_{95}: 64.61 – 98.51) isolates, followed by *pil* and *gelE* both having 83.33% (10/12, CI_{95}: 55.20 – 95.30), *fsrB* 75% (9/12, CI_{95}: 46.77– 91.11) and *ace* 50% (6/12, CI_{95}: 25.38 – 74.62) **(Fig 2 and Supplementary Figure 1).**

Fig 2: Overall prevalence of virulence genes in isolated *E. faecalis*

In bivariate analysis, a statistically significant strong correlation between the *gelE* and *fsrB* gene (Spearman correlation coefficient, ρ= 0.775; p<0.01). Additionally, a correlation was observed between the *fsrB* and *ace* gene (ρ= 0.577; p<0.05).

Regarding biofilm production, strong biofilm former isolates showed higher levels of *agg*, and *pil* genes (85.72%), whereas intermediate biofilm former isolates showed higher levels of *agg* (66.7%), *pil* (50%), *fsrB* (50%), *gelE* (50%), and *ace* (33.33%).

Antibiotic resistance profiles of *E. faecalis*

Antibiogram analysis of *E. faecalis* isolates (N = 40) revealed that, within the WHO Access antibiotic group, 90% isolates were phenotypically resistant to ampicillin, 57.5% were resistant to erythromycin, and 15% were resistant to tetracycline, Within the Watch group, 70% were resistant to rifampin, 7.5% were resistant to levofloxacin, 5% were resistant to ciprofloxacin, and 47.5% were resistant to vancomycin. From the Reserve group of antibiotics, 47.5% were resistant to linezolid (**Fig 3**). Moreover, 65% of the ampicillin-resistant isolates contained the resistance gene *bla*TEM (13/20, CI_{95}: 43.29 - 81.88). **(Supplementary Figure 2)**

Fig 3: Antibiotic resistance patterns of *E. faecalis* isolates. Legends, AMP= Ampicillin; C= Chloramphenicol; LZD= Linezolid; CIP= Ciprofloxacin; E= Erythromycin; RA= Rifampicin; TE= Tetracycline; LEV= levofloxacin and VA = Vancomycin

Statistical bivariate analysis showed that the antibiotic-resistant isolates of this study had highly significant connections with one another. Significant and positive correlation existed between resistance patterns of Vancomycin and Rifampicin (Pearson correlation coefficient, ρ=0.623; p<0.01); levofloxacin and ciprofloxacin (ρ= 0.806; p<0.01), Chloramphenicol and ciprofloxacin (ρ= 0.474; p<0.01), Chloramphenicol and levofloxacin (ρ= 0.370; p<0.05), Linezolid and

Vancomycin (ρ= 1.000; $p<0.01$), Linezolid and Rifampicin (ρ= 0.623; $p<0.01$). Linezolid and Vancomycin (ρ= 1.000; $p<0.01$), Erythromycin and Vancomycin (ρ= 0.818; $p<0.01$). Erythromycin and Rifampicin (ρ= 0.541; $p<0.01$). Erythromycin and Linezolid (ρ= 0.818; $p<0.01$). Tetracycline and Chloramphenicol (ρ= 0.546; $p<0.01$)**.**

Moreover, Strong biofilm-forming *E. faecalis* isolates exhibited higher resistance to certain antibiotics compared to intermediate and non-biofilm formers, particularly for tetracycline (42.9% vs. 0% vs. 20%) and erythromycin (57.1% vs. 33.3% vs. 100%). Ampicillin resistance was uniformly high across all biofilm categories (88.9%), whereas ciprofloxacin, levofloxacin, and chloramphenicol showed the lowest resistance rates (11.1%). Interestingly, vancomycin and linezolid resistance were more prevalent in non-biofilm formers (80%) than in strong (14.3%) or intermediate (33.3%) biofilm producers, although these differences were not statistically significant ($p>0.05$)**.**

The present study detected 12 types of resistant trends of *E. faecalis* isolated from fresh fruits surface. Of 40 PCR-positive samples, 24 (60.00%[illegible] CI_{95}: 44.6-73.65) isolates showed multidrug resistance (MDR). Furthermore, all the patterns showed resistance to a similar number of antibiotics and classes. The MAR index also fluctuated between 0.33 and 0.78 (**Table 2).**

Table 2. Occurrence of MDR *E. faecalis* isolates and multiple-antibiotic resistance (MAR

No. of pattern	Antibiotic resistance pattern	No. of antibiotics (classes)	No. of isolates	Overall, MDR isolates %	MAR index
1	**AMP, RA, LEV**	3(3)	1	24/40 (60.00%)	0.33
2	**VA, AMP, RA, LNZ, E**	5(5)	15		0.56
3	**AMP, RA, CIP, LEV, C, E, TE**	7(6)	1		0.78

4	**AMP, RA, E, TE**	4(4)	1		0.45
5	**AMP, E, TE**	3(3)	1		0.33
6	**VA, AMP, RA, LNZ, E, TE**	6(6)	1		0.67
7	**AMP, C, E, TE**	4(4)	3		0.45
8	**VA, RA, LNZ, E**	4(4)	1		

Legends, R=Resistant; I= Intermediate; S= Sensitive; AMP= Ampicillin; C= Chloramphenicol; LZD= Linezolid; CIP= Ciprofloxacin; E= Erythromycin; RA= Rifampicin; TE= Tetracycline; LEV= levofloxacin and VA = Vancomycin.

Discussion

Enterococcus faecalis is a Gram-positive bacterium that is mostly commensal, which is found in humans, animals, and a variety of habitats, and can sometimes be an opportunistic pathogen. It is one of the main causes of nosocomial infections in the world [34]. Other pathogens can acquire resistance genes from *E. faecalis* strains that are multidrug-resistant (MDR) through horizontal gene transmission [35]. The three most common infections linked to healthcare, surgical site infections, bloodstream infections, and catheter-associated urinary tract infections (CAUTI), are all significantly influenced by enterococci. One actual zoonotic pathogen that might have a major effect on human health is *E. faecalis*. Its capacity to infect humans and animals and acquire genes resistant to antibiotics makes this especially concerning [36].

Prevalence of *Enterococcus faecalis*

The overall prevalence of *E. faecalis* in this study was 61.84%; the detection rate was greater in the Seshmore and Kewatkhali area (75%), and it was particularly high in oranges (70%), apples (66.67%), and mangos (62.5%), respectively. High population density, poor vendor hygiene, using contaminated or unclean water to wash or spray on fruits to keep them fresh, cross-contamination from display surfaces and human touch, and exposure to dust and wastewater may all contribute

to the greater prevalence of *E. faecalis* in samples taken from these areas [37–39]. These factors can all contribute to the persistence and spread of *E. faecalis*.

Depending on the type of fruits and the source of the sample, similar studies have revealed different prevalence rates. For instance, a study conducted in northern Georgia found that *E. faecalis* was highly prevalent in fruits and vegetables, especially in tomatoes (33%) and radishes (91%) [6]. According to a study, the prevalence in the fruit samples was 50%, which was much lower than that in the vegetable (86.5%) and salad (82.6%) samples [40], while another study detected a prevalence of 16% in salad vegetables [41]. Differences in management practices, wash water quality, fruit handling, market and vendor cleanliness, geographic location, ambient conditions, and the regional microbial burden can all have an impact on variations in the prevalence of *E. faecalis* in raw fruits. Such fruits can be a direct cause of infection when eaten raw [42–44]. This emphasizes the necessity of better cleanliness and secure handling procedures in public marketplaces.

Biofilm-forming Capabilities of Isolated *E. faecalis*

In our study, 38.88% isolates showed strong biofilm-forming ability, 33.33% showed intermediate, and 27.78% isolates were found to be non-biofilm producers. Our study suggests that Enterococcal pathogenicity and antibiotic resistance may be increased by biofilm development in the strong biofilm-forming isolates [45]. Biofilm formation is a critical survival strategy that enables *E. faecalis* to persist in hostile environments, evade host immune responses, and resist antimicrobial treatments. A previous study found that *E. faecalis* isolated from the food samples had biofilm-forming capabilities, with 13.3% as strong biofilm-forming *E. faecalis* [46]. The capacity of *E. faecalis* isolates from fruit samples to form biofilms ranged from moderate to strong, suggesting that the bacteria can stick to fruit surfaces and possibly resist handling and storage. This tenacity

raises the possibility that consumers will become contaminated, particularly if fruits are consumed raw. As biofilm development increases bacterial survival and tolerance to antibiotics and disinfectants, even strains that seem somewhat resistant to them may still be a danger for infection. The isolates' differing abilities to build biofilms suggest that certain strains may be particularly adept at surviving on fruits; therefore, proper handling and strict hygiene are essential from farm to table.

Virulence Genes in Isolated *E. faecalis*

The main findings of this study were that *E. faecalis* isolates may frequently have multiple virulence genes. This study detected five different virulence genes in the *E. faecalis* isolated from fresh fruits surface. All the *E. faecalis* isolates from fruit samples were found to be positive for multiple virulence genes, where *agg, ace, gelE, fsrB,* and *pil* genes were detected in 91.67%, 50%, 83.33%, 75% and 83.33% of the isolates, respectively. Very few studies have investigated the presence of *E. faecalis* on fresh fruit surfaces, and even fewer have characterized their virulence profiles. In one study found that 74 potential virulence genes were found in *E. faecalis* isolates from salad samples using whole-genome sequencing, with 17–44 genes per isolate [41]. The universal presence of the adhesion genes, *ace* (100 %) and *efaA* (100%), the biofilm-associated *fsr* operon, gelatinase (*gelE*, 100%), and metalloprotease *sprE* were among the important discoveries (100%). However, a study in Korea found that 38.1% of the *gelE* gene from *E. faecalis* isolated from various food sources, which is far less than our results [47]. Other reported that, among *E. faecalis* isolates from fish, vegetables, and humans, the most frequent virulence gene was *gelE* (65.38%), followed by *ace* (51.92%), *cylA* (48.08%), *asa1* (40.38%), and *esp* (25%), while *hyl* was the least frequent gene (1.92%) [48]. The genes ace, *fsrC, gelE*, and *sprE,* which are linked to attachment and biofilm formation in *E. faecalis*, showed clear positive relationships [49]. Since

their presence on raw fruits represents a potential foodborne transmission route for pathogenic and multidrug-resistant *E. faecalis*, strict hygiene during handling, purchasing, and processing is necessary.

Antimicrobial resistance profiles of *E. faecalis*

In this study, high resistance was observed against ampicillin (90%) and rifampicin (70%), while moderate resistance was found for erythromycin (57.5%) and vancomycin (47.5%), as well as linezolid (47.5%). Lower resistance levels were recorded for tetracycline (15%), levofloxacin (7.5%), ciprofloxacin (5%), and chloramphenicol (5%). Ampicillin is one of the highly prescribed antibiotics in Bangladesh [50,51], which suggests widespread distribution in the environment and aligns with our findings. Among the twenty randomly selected Ampicillin-resistant isolates, 65% were found to be positive for *blaTEM*, suggesting the involvement of mobile resistance determinants facilitating horizontal gene transfer. The prevalence of vancomycin-resistant isolates (47.5%) on fruit surfaces is alarming due to the significant role VRE play in hospital-acquired infections. Vancomycin is a critical antibiotic used to treat infections caused by enterococci. VRE on fruits indicates that resistance genes can be acquired by environmental exposure or horizontal gene transfer, even in populations that are not directly exposed to antibiotics. This emphasizes how important it is to handle and sell fruit with extreme hygienic measures to reduce the possibility of human transmission of resistant *E. faecalis*. Studies carried out in a variety of geographical locations, including Ghana [52], Japan [53], the USA [6], and Brazil [54], have revealed that enterococci isolated from different foods have variable degrees of AMR against a variety of antibiotics. Strong positive correlations between multiple antibiotic co-resistance patterns were revealed by bivariate analysis of antibiotic-resistant *E. faecalis* from fresh fruits. These patterns included Vancomycin-Rifampicin (ρ = 0.623), Levofloxacin-Ciprofloxacin (ρ = 0.806),

Linezolid–Vancomycin (ρ = 1.000), Erythromycin–Vancomycin (ρ = 0.818), and Tetracycline–Chloramphenicol (ρ = 0.546). Furthermore, 60.00% isolates had an MDR classification, with MAR index values ranging from 0.33 to 0.78. These MDR isolates showed a variety of resistance patterns that were indicative of either significant selection pressure or antimicrobial exposure. Despite the growing concern, research on the multidrug resistance (MDR) profiles of *E. faecalis* isolated from fruit surfaces remains scarce. But similar findings were reported in one study, with 29.8% of the bacterial strains isolated from lettuce and fruits reported to be multidrug-resistant (MDR) [55]. Another study found a 67.93% prevalence of MDR in bacterial isolates recovered from fresh fruits and vegetables [56]. The presence of MDR *E. faecalis* on fruits is particularly alarming, as consumption of such contaminated fruits could facilitate the transmission of resistant strains to humans. This not only increases the risk of difficult-to-treat infections but also contributes to the potential dissemination of resistance genes within the community ecosystem.

Conclusion

To the best of our knowledge, this study is the first to report the resistance profile, virulence determinants, and biofilm-forming ability of *E. faecalis* isolated from the surface of fresh fruits sold in and around Mymensingh city, Bangladesh. The findings reveal a concerning presence of multi-drug-resistant (MDR) *E. faecalis*, capable of forming biofilms and carrying virulence genes, with resistance to critically important antibiotics such as vancomycin and linezolid. These results indicate that fresh fruits may serve as potential carriers of MDR *E. faecalis*, posing a significant risk of transmission to humans and thus posing a serious public health concern. This study highlights the urgent need for strict enforcement of hygiene practices in fruit handling, storage, and distribution. Furthermore, it underscores the importance of continuous monitoring and

research to track antimicrobial resistance in fresh produce and to protect public health in Bangladesh.

Acknowledgments

The authors would like to convey their sincere appreciation to the Department of Microbiology and Hygiene, Faculty of Veterinary Science, Bangladesh Agricultural University, Mymensingh-2202, for the significant support provided throughout this research undertaking.

Funding

The authors extend their appreciation to the Bangladesh Agricultural University Research System (BAURES) (grant No. 2024/141/BAU) for partial funding of the research work. The funders had no role in the study design, data collection, and analysis, decision to publish, or preparation of the manuscript.

Consent for publication

Not applicable

Competing interests

The authors have declared that no competing interests exist.

Data Availability Statement

Datasets will be available on request from the corresponding author on reasonable request.

Reference

1. Murray BE. The life and times of the enterococcus. Clin Microbiol Rev. 1990;3: 46–65.
2. Hammerum AM, et al. Enterococci of animal origin and their significance for public health. Clin Microbiol Infect. 2012;18:619-25

3. Bayer AS, Seidel JS, Yoshikawa TT, Anthony BF, Guze LB. Group D Enterococcal Meningitis: Clinical and Therapeutic Considerations with Report of Three Cases and Review of the Literature. Arch Intern Med. 1976;136: 883–886.

4. McDonald LC, Kuehnert MJ, Tenover FC, Jarvis WR. Vancomycin-resistant enterococci outside the health-care setting: prevalence, sources, and public health implications. Emerg Infect Dis. 1997;3: 311.

5. Leff JW, Fierer N. Bacterial Communities Associated with the Surfaces of Fresh Fruits and Vegetables. PLoS One. 2013;8: e59310.

6. Balali GI, Yar DD, Afua Dela VG, Adjei-Kusi P. Microbial Contamination, an Increasing Threat to the Consumption of Fresh Fruits and Vegetables in Today's World. Int J Microbiol. 2020;2020: 3029295.

7. McGowan LL, Jackson CR, Barrett JB, Hiott LM, Fedorka-Cray PJ. Prevalence and antimicrobial resistance of enterococci isolated from retail fruits, vegetables, and meats. J Food Prot. 2006;69: 2976–2982.

8. Tenea GN, Reyes P, Molina D, Ortega C. Pathogenic Microorganisms Linked to Fresh Fruits and Juices Purchased at Low-Cost Markets in Ecuador, Potential Carriers of Antibiotic Resistance. Antibiotics. 2023;12: 236.

9. Yi L, Chen S, Li G, Ren J, Zhou R, Zeng K. Prevalence of antibiotic-resistant pathogens in online fresh-cut fruit from Chongqing, China, and controlling Enterococcus faecalis by bacteriocin GF-15. LWT. 2022;165: 113678.

10. Oprea SF, Zervos MJ. Enterococcus and its Association with Foodborne Illness. Foodborne Diseases. Humana Press; 2007; 157–174.

11. Fahim NAI, Rana ML, Hasan MAE, Salam S, Masud RI, Huda N, et al. Biofilms and antibiotic resistance profile of Enterococcus faecalis in selected dairy cattle farm environments in Bangladesh. PLoS One. 2025;20: e0323667.

12. McGowan LL, Jackson CR, Barrett JB, Hiott LM, Fedorka-Cray PJ. Prevalence and antimicrobial resistance of enterococci isolated from retail fruits, vegetables, and meats. J Food Prot. 2006;69: 2976–2982.

13. Finley RL, Collignon P, Larsson DG, McEwen SA, Li XZ, Gaze WH, et al. The scourge of antibiotic resistance: the important role of the environment. Clin Infect Dis. 2013;57:704-10.

14. Witte W. Ecological impact of antibiotic use in animals on different complex microflora: Environment. Int J Antimicrob Agents. 2000;14: 321–325.

15. Fahim NAI, Masud RI, Salam S, Hasan MAE, Rahman AMMT, Punom SA, et al. Role of Enterococcus in spreading antimicrobial resistance genes and its public health significance. Ger J of Vet Res. 2025;5: 95–112.

16. Sirichoat A, Flórez AB, Vázquez L, Buppasiri P, Panya M, Lulitanond V, et al. Antibiotic Resistance-Susceptibility Profiles of Enterococcus faecalis and Streptococcus spp. From the Human Vagina, and Genome Analysis of the Genetic Basis of Intrinsic and Acquired Resistances. Front Microbiol. 2020;11:1438.

17. Sadanandan B, Yogendraiah KM. Enterococcus faecalis Biofilm: A Clinical and Environmental Hazard. Medical Sciences Forum. 2025;35: 5.

18. Hakim TA, Zaki BM, Mohamed DA, Blasdel B, Gad MA, Fayez MS, et al. Novel strategies for vancomycin-resistant Enterococcus faecalis biofilm control: bacteriophage (vB_EfaS_ZC1), propolis, and their combined effects in an ex vivo endodontic model. Ann Clin Microbiol Antimicrob. 2025;24:24.

19. Mubarak AG, El-Zamkan MA, Younis W, Saleh SO, Abd-Elhafeez HH, Yoseef AG. Phenotypic and genotypic characterization of Enterococcus faecalis and Enterococcus faecium isolated from fish, vegetables, and humans. Sci Rep. 2024;14:21741.

20. Boeder AM, Spiller F, Carlstrom M, Izídio GS. Enterococcus faecalis: implications for host health. World J Microbiol Biotechnol. 2024;40:190.

21. Azzam A, Elkafas H, Khaled H, Ashraf A, Yousef M, Elkashef AA, et al. Prevalence of Vancomycin-resistant enterococci (VRE) in Egypt (2010-2022): a systematic review and meta-analysis. J Egypt Public Health Assoc. 2023;98:8.

22. Ballah FM, Islam MS, Rana ML, Ferdous FB, Ahmed R, Pramanik PK, et al. Phenotypic and Genotypic Detection of Biofilm-Forming Staphylococcus aureus from Different Food Sources in Bangladesh. Biology. 2022;11: 949.

23. Sarker MAR, Haque MM, Rifa RA, Ema FA, Islam MA, Khatun MM. Isolation and identification of bacteria from fresh guava (Psidium guajava) sold at local markets in Mymensingh and their antibiogram profile. Vet World. 2018;11: 1145–1149.

24. Salam S, Fahim NAI, Ashraf MdN, Masud RI, Jahan R, Antor MdTH, et al. Virulence profiling, biofilm formation, and antimicrobial resistance pattern of Enterococci associated with poultry and livestock feeds. J of Appl Poult Res. 2025; 100590.

25. Fahim NAI, Rana ML, Hasan MAE, Salam S, Masud RI, Huda N, et al. Biofilms and antibiotic resistance profile of Enterococcus faecalis in selected dairy cattle farm environments in Bangladesh. PLoS One. 2025;20.

26. Dutka-Malen S, Evers S, Courvalin P. Detection of glycopeptide resistance genotypes and identification to the species level of clinically relevant enterococci by PCR. J Clin Microbiol. 1995;33: 24–27.

27. Hashem YA, Amin HM, Essam TM, Yassin AS, Aziz RK. Biofilm formation in enterococci: Genotype-phenotype correlations and inhibition by vancomycin. Sci Rep. 2017;7: 1–12.

28. Randall LP, Cooles SW, Osborn MK, Piddock LJV, Woodward MJ. Antibiotic resistance genes, integrons and multiple antibiotic resistance in thirty-five serotypes of Salmonella enterica isolated from humans and animals in the UK. Journal of Antimicrobial Chemotherapy. 2004;53: 208–216.

29. Zheng JX, Bai B, Lin ZW, Pu ZY, Yao WM, Chen Z, et al. Characterization of biofilm formation by Enterococcus faecalis isolates derived from urinary tract infections in China. J Med Microbiol. 2018;67: 60–67.

30. de Souza EL, Meira QGS, Barbosa I de M, Athayde AJAA, da Conceição ML, de Siqueira JP. Biofilm formation by Staphylococcus aureus from food contact surfaces in a meat-

based broth and sensitivity to sanitizers. Brazilian Journal of Microbiology. 2014;45: 67–75.

31. CLSI. Performance Standards for Antimicrobial Susceptibility Testing. 34th ed. CLSI supplement M100. Clinical and Laboratory Standards Institute; 2024.

32. Sweeney MT, Lubbers B V., Schwarz S, Watts JL. Applying definitions for multidrug resistance, extensive drug resistance, and pandrug resistance to clinically significant livestock and companion animal bacterial pathogens. Journal of Antimicrobial Chemotherapy. 2018;73: 1460–1463.

33. Krumperman PH. Multiple antibiotic resistance indexing of Escherichia coli to identify high-risk sources of fecal contamination of foods. Appl Environ Microbiol. 1983;46: 165–170.

34. Brown LD, Cai TT, Das Gupta A. Interval estimation for a binomial proportion. Statistical Science. 2001;16: 101–117.

35. Upadhyaya GPM, Ravikumar KL, Umapathy BL. Review of virulence factors of enterococcus : AAAn emerging nosocomial pathogen. Indian J Med Microbiol. 2009;27: 301–305.

36. Guan L, Beig M, Wang L, Navidifar T, Moradi S, Motallebi Tabaei F, et al. Global status of antimicrobial resistance in clinical Enterococcus faecalis isolates: systematic review and meta-analysis. Ann Clin Microbiol Antimicrob. 2024;23.

37. Abat C, Huart M, Garcia V, Dubourg G, Raoult D. Enterococcus faecalis urinary-tract infections: Do they have a zoonotic origin? Journal of Infection. W.B. Saunders. 2016; 305–313.

38. Khanal AR, Timilsina RH, Sharma B, Pokharel B, Aryal R. Contaminated Water and an Indication of Risk: Examining Microbial Contamination in the Water Used by Consumers and Commercial Growers in Fresh Produce Systems in Nepal. J Food Prot. 2024;87.

39. Bartz FE, Lickness JS, Heredia N, de Aceituno AF, Newman KL, Hodge DW, et al. Contamination of fresh produce by microbial indicators on farms and in packing facilities: Elucidation of environmental routes. Appl Environ Microbiol. 2017;83.

40. Demisie KN, Melese DM. Assessment of bacterial and parasitic contamination of fruits gathered from specific local markets in Addis Ababa, Ethiopia. Front Sustain Food Syst. 2024;8: 1402898.

41. Peng Z, Hu Y, Ye Z, Deng J, Yang D, Xu J, et al. One Health Approach of Enterococcal Population Structure and Antibacterial Resistance Along the Food Chain - Four PLADs, China, 2015-2022. China CDC Wkly. 2024;6:1223-1231.

42. Habib I, Khan M, Lakshmi GB, Mohamed MYI, Ghazawi A, Al-Rifai RH. Salad Vegetables as a Reservoir of Antimicrobial-Resistant Enterococcus: Exploring Diversity, Resistome, Virulence, and Plasmid Dynamics. Foods. 2025;14: 1150.

43. Rahman M, Alam MU, Luies SK, Kamal A, Ferdous S, Lin A, et al. Contamination of fresh produce with antibiotic-resistant bacteria and associated risks to human health: A scoping review. Int J Environ Res Public Health. 2022;19: 360.

44. Akter S, Zuhura FT, Bhuiyan MNI, Sultana N. Assessment of bacterial contamination in fresh produce marketed in Sadarghat shops in Dhaka city. Bangladesh Journal of Scientific and Industrial Research. 2025;60: 115–124.

45. Khalil MA, Alorabi JA, Al-Otaibi LM, Ali SS, Elsilk SE. Antibiotic Resistance and Biofilm Formation in Enterococcus spp. Isolated from Urinary Tract Infections. Pathogens. 2023;12: 34.

46. Anderson AC, Jonas D, Huber I, Karygianni L, Wölber J, Hellwig E, et al. Enterococcus faecalis from food, clinical specimens, and oral sites: Prevalence of virulence factors in association with biofilm formation. Front Microbiol. 2016;6: 169340.

47. Kim NH, Kim HW, Park SM, Seo GH, Cho TJ, Yu HR, et al. Virulence patterns and prevalence of seven Enterococcus species isolated from meats and leafy vegetables in South Korea. Food Control. 2020;108: 106867.

48. Mubarak AG, El-Zamkan MA, Younis W, Saleh SO, Abd-Elhafeez HH, Yoseef AG. Phenotypic and genotypic characterization of Enterococcus faecalis and Enterococcus faecium isolated from fish, vegetables, and humans. Sci Rep. 2024;14: 1–17.

49. Bag MAS, Arif M, Riaz S, Khan MSR, Islam MS, Punom SA, et al. Antimicrobial Resistance, Virulence Profiles, and Public Health Significance of Enterococcus faecalis Isolated from Clinical Mastitis of Cattle in Bangladesh. Biomed Res Int. 2022;2022: 8101866.

50. Fahad BM, Matin A, Shill MC, Asish KD. Antibiotic usage at a primary health care unit in Bangladesh. Australasian Medical Journal. 2010;3: 414–421.

51. Bibi S, Chisti MJ, Akram F, Pietroni MAC. Ampicillin and gentamicin are a useful first-line combination for the management of sepsis in under-five children at an urban hospital in Bangladesh. J Health Popul Nutr. 2012;30: 487–490.

52. Amuasi GR, Dsani E, Owusu-Nyantakyi C, Owusu FA, Mohktar Q, Nilsson P, et al. Enterococcus species: insights into antimicrobial resistance and whole-genome features of isolates recovered from livestock and raw meat in Ghana. Front Microbiol. 2023;14: 1254896.

53. Yamagami Y, Asao M, Takahashi A, Hashimoto Y, Okuyama N, Arai E, et al. Prevalence and antimicrobial resistance of Enterococcus spp. isolated from animal feed in Japan. Front Vet Sci. 2024;10.

54. Riboldi GP, Frazzon J, D'Azevedo PA, Frazzon APG. Antimicrobial resistance profile of Enterococcus spp isolated from food in Southern Brazil. Brazilian Journal of Microbiology. 2009;40: 125.

55. Carvalheira A, Silva J, Teixeira P. Lettuce and fruits as a source of multidrug resistant Acinetobacter spp. Food Microbiol. 2017;64: 119–125.

56. Olanbiwoninu A, Awotundun T, Olayiwola J, Somorin Y. Antimicrobial-resistant pathogens in fruits and vegetables from retail and home gardens. Sustainable Microbiology. 2024;1.

Supporting information

Supplementary Figure 1: PCR amplification of primers used to detect virulence gene (a) PCR detection of *agg* gene with amplicon size 413 bp; (b) PCR detection of *gelE* gene with amplicon size 704 bp ; (c) PCR detection of *pil* gene with amplicon size 620 bp; (d) PCR detection of *ace* gene with amplicon size 615 bp (Left); and *fsrB*, gene with amplicon size 428 bp (Right) . In all cases, L: 100 bp DNA ladder; NC: negative control; PC: positive control).

Supplementary Figure 2: PCR amplification of primers used to detect resistance gene, PCR detection of *blaTEM* gene with amplicon size 793 bp; L: 100 bp DNA ladder; NC: negative control; PC: positive control.

Figure Click here to access/download;Figure;Fig. 1.jpg

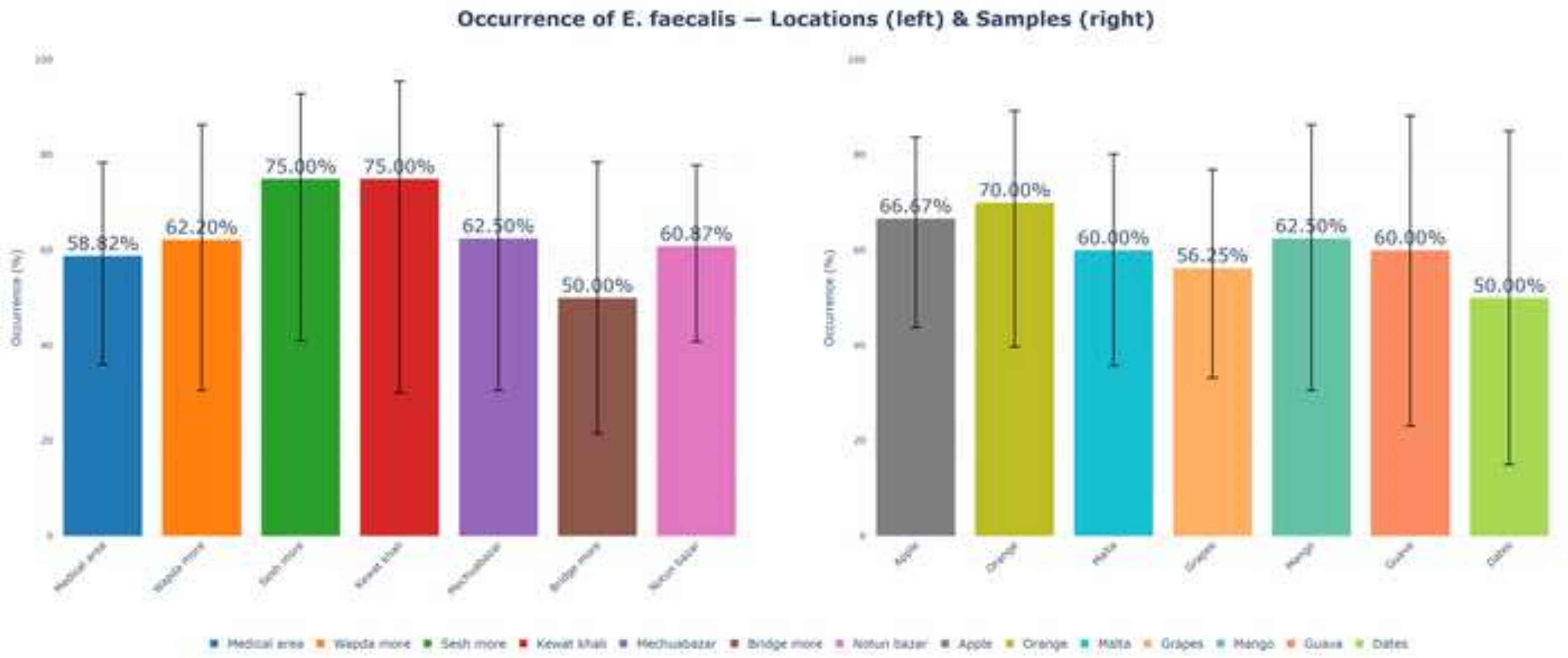

Figure

Click here to access/download;Figure;Fig. 2.jpeg

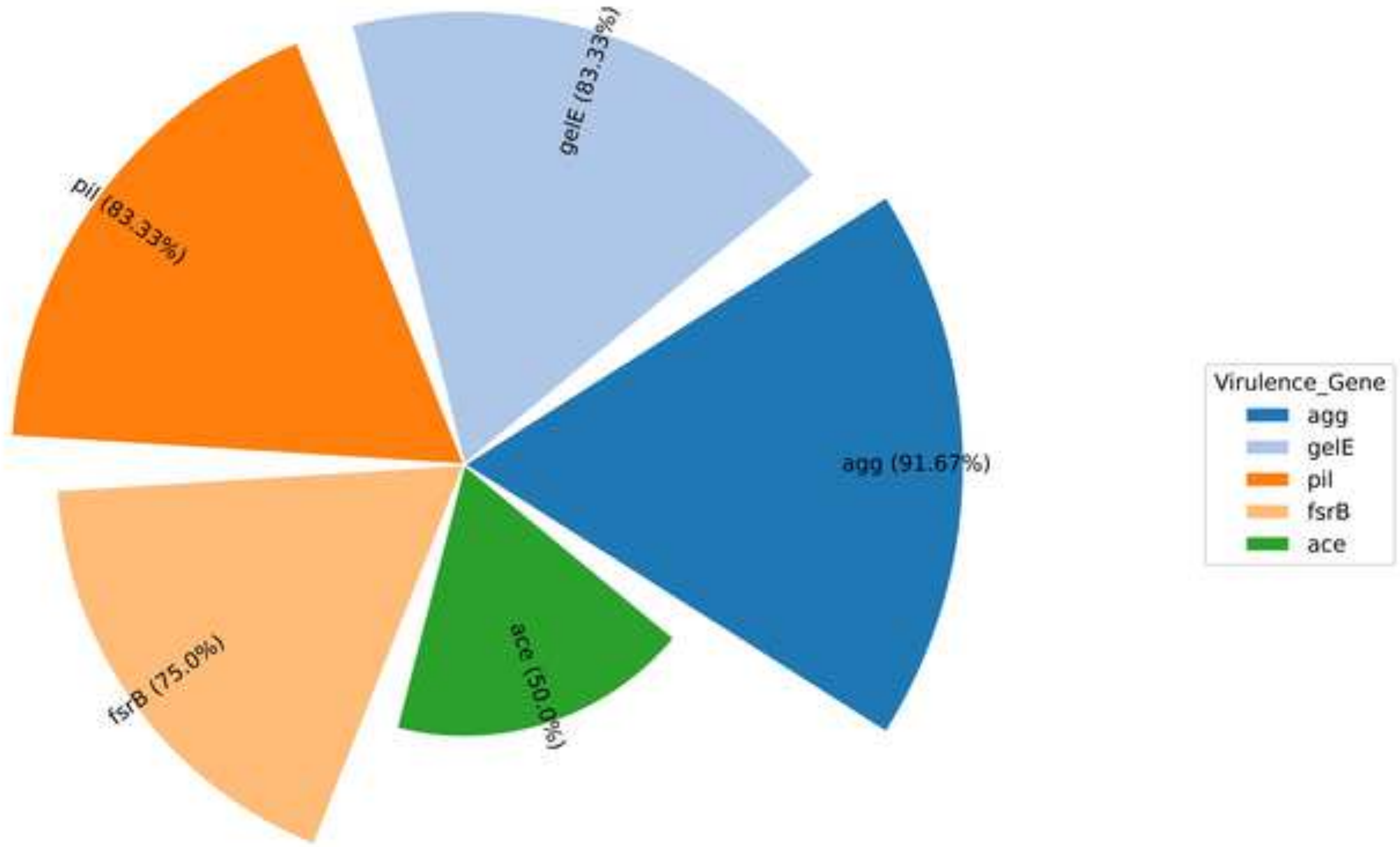

Figure

Click here to access/download;Figure;Fig. 3.jpeg

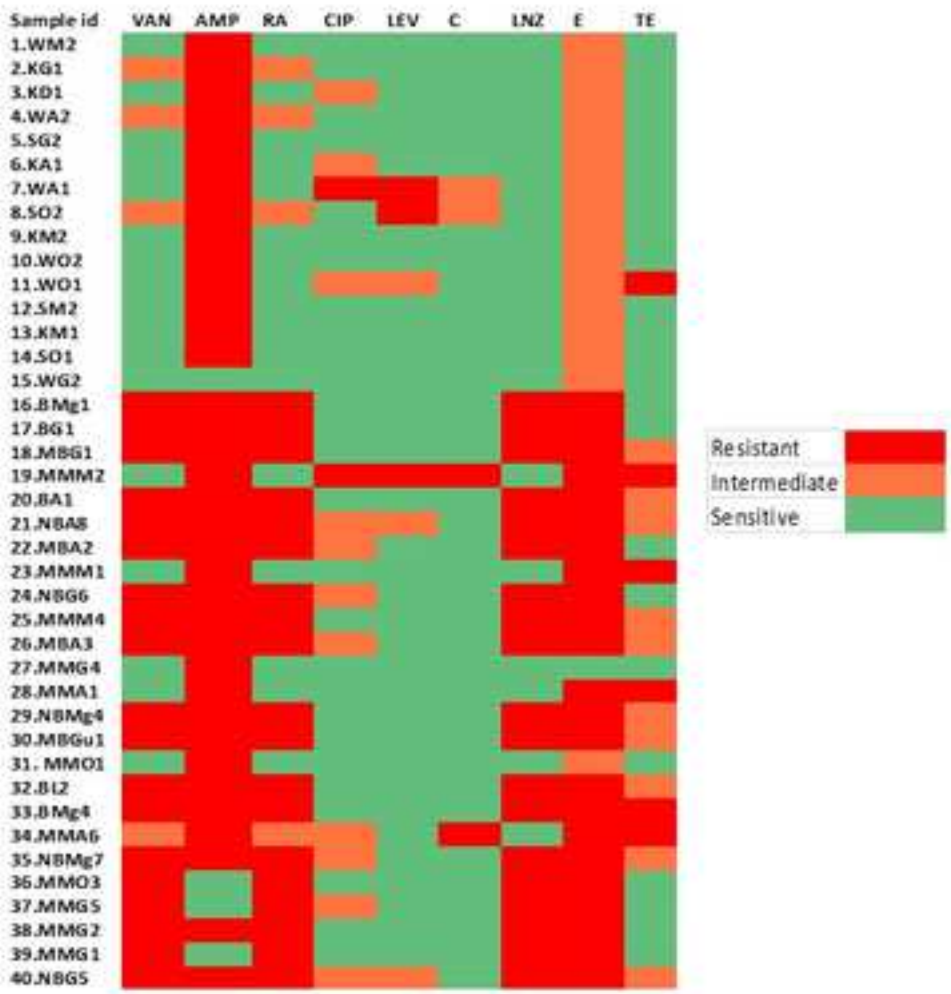

Click here to access/download
Supporting Information
Supplementary Figure.docx
