# Supplementary material for: Fresh fruit surface: A potential source for multidrug-resistant biofilm-forming Enterococcus faecalis having potential public health significance
Source: PLoS One. 2026 Aug 5;21(8):e0355410. doi: 10.1371/journal.pone.0355410 (PMC13440884; doi:10.1371/journal.pone.0355410)
Supplement: Supplementary file 2 [file pone.0355410.s001.docx]

**Supplementary Figure 1**

PCR based amplification of various virulence genes from *Enterococcus faecalis*

**Supplementary Figure 1: PCR based amplification of virulence gene** from *E. faecalis* isolated from fresh fruit samples (a) PCR detection of *agg* gene with amplicon size 413 bp; (b) PCR detection of *gelE* gene with amplicon size 704 bp ; (c) PCR detection of *pil* gene with amplicon size 620 bp; (d) PCR detection of *ace* gene with amplicon size 615 bp (Left); and *fsrB,* gene with amplicon size 428 bp (Right). In all cases, L: 100 bp DNA ladder; NC: negative control; PC: positive control.
