# Supplementary material for: Fresh fruit surface: A potential source for multidrug-resistant biofilm-forming Enterococcus faecalis having potential public health significance
Source: PLoS One. 2026 Aug 5;21(8):e0355410. doi: 10.1371/journal.pone.0355410 (PMC13440884; doi:10.1371/journal.pone.0355410)
Supplement: Supplementary file 3 [file pone.0355410.s002.docx]

**Supplementary Figure 2**

PCR-based amplification of *blaTEM* gene from *E. faecalis*  isolated from fresh fruit samples

**Supplementary Figure 2: PCR-based amplification of the resistance gene**, PCR detection of the *blaTEM* gene with an amplicon size of 793 bp; L: 100 bp DNA ladder; NC: negative control; PC: positive control.
