# Supplementary material for: Fresh fruit surface: A potential source for multidrug-resistant biofilm-forming Enterococcus faecalis having potential public health significance
Source: PLoS One. 2026 Aug 5;21(8):e0355410. doi: 10.1371/journal.pone.0355410 (PMC13440884; doi:10.1371/journal.pone.0355410)
Supplement: Supplementary file 4 [file pone.0355410.s003.docx]

**Supplementary Table 1: Correlation between two virulence genes in *Enterococcus faecalis* isolated from various fresh fruit samples**

|  | | *agg* | *pil* | *gelE* | *ace* | *fsrB* |
| --- | --- | --- | --- | --- | --- | --- |
| *agg* | Pearson Correlation | 1 |  |  |  |  |
|  | Sig. (2-tailed) |  |  |  |  |  |
| *pil* | Pearson Correlation | -0.135 | 1 |  |  |  |
|  | Sig. (2-tailed) | 0.676 |  |  |  |  |
| *gelE* | Pearson Correlation | -0.135 | 0.400 | 1 |  |  |
|  | Sig. (2-tailed) | 0.676 | 0.198 |  |  |  |
| *ace* | Pearson Correlation | -0.302 | 0.000 | 0.447 | 1 |  |
|  | Sig. (2-tailed) | 0.341 | 1.000 | 0.145 |  |  |
| *fsrB* | Pearson Correlation | -0.174 | 0.258 | .775^**^ | .577^*^ | 1 |
|  | Sig. (2-tailed) | 0.588 | 0.418 | 0.003 | 0.049 |  |
| **. Correlation is significant at the 0.01 level (2-tailed). | | | | | | |
| *. Correlation is significant at the 0.05 level (2-tailed). | | | | | | |

**Legends,** R= Resistant; I= Intermediate; S= Sensitive; AMP= Ampicillin; C= Chloramphenicol; LZD= Linezolid; CIP= Ciprofloxacin; E= Erythromycin; RA= Rifampicin; TE= Tetracycline; LEV= levofloxacin and VA = Vancomycin.
