# Supplementary material for: Fresh fruit surface: A potential source for multidrug-resistant biofilm-forming Enterococcus faecalis having potential public health significance
Source: PLoS One. 2026 Aug 5;21(8):e0355410. doi: 10.1371/journal.pone.0355410 (PMC13440884; doi:10.1371/journal.pone.0355410)
Supplement: Supplementary file 5 [file pone.0355410.s004.docx]

**Supplementary Table 2: Antibiotic resistance profile of *Enterococcus faecalis* isolated from various fresh fruit samples**

| **Sample Source** | **ID** | **VA** | **AMP** | **RA** | **CIP** | **LEV** | **C** | **LZD** | **E** | **TET** |
| --- | --- | --- | --- | --- | --- | --- | --- | --- | --- | --- |
| Malta | WM2 | S | R | R | S | S | S | S | I | S |
|  | KM2 | S | R | I | S | S | S | S | I | S |
|  | SM2 | S | R | I | S | S | S | S | I | S |
|  | KM1 | S | R | I | S | S | S | S | I | S |
|  | MMM2 | S | R | R | R | R | R | S | R | R |
|  | BA1 | R | R | R | S | S | S | R | R | I |
|  | MMM1 | S | R | R | S | S | S | S | R | R |
|  | MMM4 | R | R | R | S | S | S | R | R | I |
| Grape | MMG5 | R | S | R | I | S | S | R | R | S |
|  | MMG2 | R | R | R | S | S | S | R | R | S |
|  | MMG1 | R | S | R | S | S | S | R | R | S |
|  | NBG5 | R | R | R | I | I | S | R | R | I |
|  | WG2 | S | S | S | S | S | S | S | I | S |
|  | BG1 | R | R | R | S | S | S | R | R | S |
|  | MBG1 | R | R | R | S | S | S | R | R | I |
|  | NBG6 | R | R | R | I | S | S | R | R | S |
|  | MMG4 | S | R | S | S | S | S | S | S | S |
|  | KG1 | I | R | R | S | S | S | S | I | S |
|  | SG2 | S | R | R | S | S | S | S | I | S |
| Orange | MMO3 | R | S | R | S | S | S | R | R | S |
|  | SO2 | I | R | R | S | R | I | S | I | S |
|  | WO2 | S | R | I | S | S | S | S | I | S |
|  | WO1 | S | R | I | I | I | S | S | I | R |
|  | SO1 | S | R | R | S | S | S | S | I | S |
|  | MMO1 | S | R | I | S | S | S | S | I | S |
| Dates | KD1 | S | R | R | I | S | S | S | I | S |
| Guava | MBGu1 | R | R | R | S | S | S | R | R | I |
|  | BGu2 | R | R | R | S | S | S | R | R | I |
| Apple | WA2 | I | R | R | S | S | S | S | I | S |
|  | KA1 | S | R | I | I | S | S | S | I | S |
|  | WA1 | S | R | I | R | R | I | S | I | S |
|  | NBA8 | R | R | R | I | I | S | R | R | I |
|  | MBA2 | R | R | R | I | S | S | R | R | S |
|  | MBA3 | R | R | R | I | S | S | R | R | I |
|  | MMA1 | S | R | S | S | S | S | S | R | R |
|  | MMA6 | I | R | I | I | S | R | S | R | R |
| Mango | NBMg4 | R | R | R | S | S | S | R | R | I |
|  | BMg4 | R | R | R | S | S | S | R | R | R |
|  | NBMg7 | R | R | R | I | S | S | R | R | I |
|  | BMg1 | R | R | R | S | S | S | R | R | S |

**Legends,** AMP= Ampicillin; C= Chloramphenicol; LZD= Linezolid; CIP= Ciprofloxacin; E= Erythromycin; RA= Rifampicin; TE= Tetracycline; LEV= levofloxacin and VA = Vancomycin.
