# Supplementary material for: Fresh fruit surface: A potential source for multidrug-resistant biofilm-forming Enterococcus faecalis having potential public health significance
Source: PLoS One. 2026 Aug 5;21(8):e0355410. doi: 10.1371/journal.pone.0355410 (PMC13440884; doi:10.1371/journal.pone.0355410)
Supplement: Supplementary file 6 [file pone.0355410.s005.docx]

**Supplementary Table 3: Correlation between two resistant antibiotics in *Enterococcus faecalis* isolated from various fresh fruit samples**

|  | | VA | AMP | RA | CIP | LEV | C30 | LNZ | E | TE |
| --- | --- | --- | --- | --- | --- | --- | --- | --- | --- | --- |
| VA | Pearson Correlation | 1 |  |  |  |  |  |  |  |  |
|  | Sig. (2-tailed) |  |  |  |  |  |  |  |  |  |
| AMP | Pearson Correlation | -0.184 | 1 |  |  |  |  |  |  |  |
|  | Sig. (2-tailed) | 0.257 |  |  |  |  |  |  |  |  |
| RA | Pearson Correlation | .623^**^ | -0.036 | 1 |  |  |  |  |  |  |
|  | Sig. (2-tailed) | 0.000 | 0.824 |  |  |  |  |  |  |  |
| CIP | Pearson Correlation | -0.218 | 0.076 | -0.100 | 1 |  |  |  |  |  |
|  | Sig. (2-tailed) | 0.176 | 0.639 | 0.539 |  |  |  |  |  |  |
| LEV | Pearson Correlation | -0.271 | 0.095 | -0.021 | .806^**^ | 1 |  |  |  |  |
|  | Sig. (2-tailed) | 0.091 | 0.560 | 0.899 | 0.000 |  |  |  |  |  |
| C | Pearson Correlation | -0.218 | 0.076 | -0.100 | .474^**^ | .370^*^ | 1 |  |  |  |
|  | Sig. (2-tailed) | 0.176 | 0.639 | 0.539 | 0.002 | 0.019 |  |  |  |  |
| LNZ | Pearson Correlation | 1.000^**^ | -0.184 | .623^**^ | -0.218 | -0.271 | -0.218 | 1 |  |  |
|  | Sig. (2-tailed) | 0.000 | 0.257 | 0.000 | 0.176 | 0.091 | 0.176 |  |  |  |
| E | Pearson Correlation | .818^**^ | -0.118 | .541^**^ | -0.035 | -0.139 | 0.197 | .818^**^ | 1 |  |
|  | Sig. (2-tailed) | 0.000 | 0.468 | 0.000 | 0.831 | 0.392 | 0.223 | 0.000 |  |  |
| TE | Pearson Correlation | -0.259 | 0.140 | -0.183 | 0.225 | 0.146 | .546^**^ | -0.259 | 0.220 | 1 |
|  | Sig. (2-tailed) | 0.106 | 0.389 | 0.257 | 0.163 | 0.368 | 0.000 | 0.106 | 0.173 |  |
| **. Correlation is significant at the 0.01 level (2-tailed). | | | | | | | | | | |
| *. Correlation is significant at the 0.05 level (2-tailed). | | | | | | | | | | |

**Legends,** R= Resistant; I= Intermediate; S= Sensitive; AMP= Ampicillin; C= Chloramphenicol; LZD= Linezolid; CIP= Ciprofloxacin; E= Erythromycin; RA= Rifampicin; TE= Tetracycline; LEV= levofloxacin and VA = Vancomycin.
