# Supplementary material for: Fresh fruit surface: A potential source for multidrug-resistant biofilm-forming Enterococcus faecalis having potential public health significance
Source: PLoS One. 2026 Aug 5;21(8):e0355410. doi: 10.1371/journal.pone.0355410 (PMC13440884; doi:10.1371/journal.pone.0355410)
Supplement: Supplementary file 7 [file pone.0355410.s007.docx]

Reviewer #1: The manuscript described molecular detection and characterization of MDR Enterococcus faecalis associated with fresh fruit surface sold in Mymensingh area. In addition to AMR, biofilm formation was also observed in this study. Considering it as the first such study in Bangladesh and the public health importance of biofilm forming MDR E. faecalis the study carries significant and crucial message for the food sellers/vendors, consumers and policy makers.

**Specific Comment:**

**1. How much DNA was used in each PCR as template?**

**Response:** Thank you. In line 128, it is mentioned that we used 50 to 60 ng of DNA as template.

**2. Congo Red assay was used for biofilm detection. Describe the principle/mechanism of Cogo Red test.**

**Response:** Thank you. Now, we have updated the manuscript with adequate information from line 139 to line 142.

**3. Several virulence genes were used. Please mention the functions of these genes; many are not mentioned in the manuscript.**

**Response:** Thank you. We have updated the gene functions in line 149 to line 150.

**4. What was the rationale for selecting those 9 antibiotics in the AST?**

**Response:** Thank you. We have mentioned the rationale for selecting those particular antibiotics in lines 155 to 158.

**5. Please remove the CI from the values, e.g., in line 180 instead of 47 (61.84%, CI95: 50.60–71.94), simply write 47 (61.84%)**

**Response:** Thank you. We have now updated that information.

**6. There are different types of fruits available in the market. Why were these particular fruits were selected?**

**Response:** Thank you for your query. We selected these fruits because they are available year-round. Most fruits in Bangladesh are seasonal. Some of them have a short shelf life, and some are not suitable for patients. As we selected shops around medical entrances, we considered fruits that are available year-round and are frequently purchased.

**7. Line 2412, Moreover,** **Strong biofilm-forming E. faecalis isolates exhibited higher resistance to certain antibiotics compared to intermediate and non-biofilm formers, explain this observed phenomenon.**
**Response:** Thank you for your suggestion. We have added an explanation in the Discussion section regarding the higher antibiotic resistance observed in strong biofilm-forming *E. faecalis* isolates. See Line: 296-298
**8. Line 252, Furthermore, all the patterns showed resistance to a similar number of antibiotics and classes. Any explanation why similar patterns were observed??**

**Response:** Thank you for your insightful observation. We have removed the line stating that “all the patterns showed resistance to a similar number of antibiotics and classes” as it was redundant and did not add meaningful interpretation.

**Reviewer #2:**

The manuscript entitlted ‘’ Fresh fruit surface: a potential source for multidrug-resistant biofilm-forming Enterococcus faecalis having potential public health significance’’ presented a well-designed and timely investigation into the occurrence, AMR, biofilm formation and virulence gene profiles of E. faecalis from fresh fruit surfaces in Bangladesh. The study addressed an important gap in food safety and One Health research, particularly in low- and middle income country where surveillance data on antimicrobial-resistant bacteria in fresh produce remain scarce.

The methodology is clearly described, results are logically structured, and discussion is well contextualized within the existing literature. Overall, the manuscript makes a valuable contribution to understanding the public health implication of antimicrobial-resistant enterococci in the food chain.

Besides its novelty and strength, several limitation should be acknowledged more explicitly to make it scientifically sound.

**Limitations and Suggestions**

**1. This study analyzed 76 fruit samples of different types exclusively from Mymensingh city, which is not adequate for an exploratory investigation, and restricts generalizability. Moreover, seasonal variation was not explicitly assessed. Thus, results should be interpreted as region-specific baseline data rather than national prevalence estimates.**

**Response:** We thank the reviewer for this observation. Although our study included 76 fruit samples from Mymensingh city, it provides new insights into antibiotic-resistant, biofilm-forming *E. faecalis* on fresh fruits in Bangladesh. These results are meant as region-specific baseline data rather than national prevalence estimates, so we selected a few samples, e.g., 76.

**2. Only 18 isolates were tested for biofilm formation and 12 isolates for virulence gene profiling, despite 47 PCR-confirmed isolates. The subsampling approach is acceptable for preliminary characterization but limits inferential depth. The authors are suggested to examine all the 47 isolates to make the result scientifically robust.**

**Response:** We thank the reviewer for this valuable suggestion. While we agree that analyzing all 47 PCR-confirmed isolates would strengthen the study, due to limited resources and reagent availability, we were only able to perform biofilm formation assays on 18 isolates and virulence gene profiling on 12 isolates. We have added this as our limitation at the end of the discussion.

**3. Biofilm formation was assessed using only the Congo Red Agar (CRA) method, which is qualitative and phenotype dependent. CRA provides rapid screening but may underestimate or overestimate true biofilm biomass.**

**Response:** We thank the reviewer for this important observation. We used the Congo Red Agar (CRA) method as a rapid and simple screening tool for biofilm formation. We acknowledge that CRA is qualitative and phenotype-dependent and may not fully reflect true biofilm biomass; however, it was suitable for preliminary characterization within the resource constraints of our study. We have added this as our limitation at the end of the discussion.

**4. Among resistance genes only** **blaTEM was screened. Key enterococcal resistance genes (e.g. vanA, vanB, ermB tetM, optrA, postA) were not investigated, despite high phenotypic resistance to vancomycin and linezolid. Phenotype-genotype discordance cannot be fully resolved without broader resistance gene profiling.**

**Response:** We thank the reviewer for this suggestion. Due to resource limitations, we screened only for the *blaTEM* gene, and we acknowledge that including additional resistance genes would provide a more complete genotype-phenotype correlation. We have added this as our limitation at the end of the discussion.
**5. The study relies on PCR-based detection rather than whole-genome sequencing. Consequently, clonal relationship, plasmid content, mobile genetic elements, and resistome structure remain unexplored. WGS in recommended for future studies to confirm transmission dynamics and One Health linkages.**

**Response:** We thank the reviewer for this insightful suggestion. In this study, we relied on PCR-based detection due to resource constraints, and we acknowledge that whole-genome sequencing (WGS) would provide deeper insights into clonal relationships, plasmid content, mobile genetic elements, and the overall resistome. Due to the funding crisis, we did not perform WGS. But we plan to incorporate WGS in our future work to better understand transmission dynamics and One Health linkages of *E. faecalis* from fresh fruits. We have added this as our limitation at the end of the discussion.

**Reviewer #3:**

Dear Editor,

This manuscript introduces a unique concept in an area that is both intriguing and important for medical research. The topic is timely and is likely to engage the journal's readers. Additionally, the manuscript is well-written, presenting ideas clearly and logically.

Dear Authors,

You are doing excellent work, and this manuscript reflects a great deal of effort and thoughtful research. The study is promising and contributes meaningfully to the field. However, there are a few important points that require further clarification and revision to enhance the overall quality and impact of the manuscript.

**Line 44: The Introduction covers several important themes (enterococci biology, public health relevance, fruit contamination, and antibiotic resistance). However, the narrative would benefit from improved coherence and a clearer logical flow. The authors are encouraged to better integrate these topics, explicitly linking Enterococcus faecalis ecology with fruit-associated transmission and public health implications.**

**Response:** Thank you for your suggestion. We have revised the introduction to make it clearer and more coherent.

**Line 46: It is preferred to use the following reference to support your updated citations: Alduhaidhawi AHM, AlHuchaimi SN, Al-Mayah TA, Al-Ouqaili MTS, Alkafaas SS, Muthupandian S, Saki M. Prevalence of CRISPR-Cas Systems and Their Possible Association with Antibiotic Resistance in Enterococcus faecalis and Enterococcus faecium Collected from Hospital Wastewater. Infect Drug Resist. 2022 Mar 19;15:1143-1154. Doi: 10.2147/IDR.S358248. PMID: 35340673; PMCID: PMC8942119.**

**Response:** Thank you for your suggestion. We have now updated the line to include a new reference. Reference 1.

**Statements suggesting that consumption of fresh fruits may increase the “baseline level of antibiotic resistance” in humans (lines 69–71) are potentially overstated. These claims should be more carefully qualified and supported with stronger evidence or rephrased to avoid causal implications not fully substantiated by current data.**

**Response:** Thank you for your concern and suggestion. We have now modified the sentence. See lines: 71-72

**Lines 76–79 repeat nearly identical statements regarding the intrinsic resistance mechanisms of E. faecalis. This redundancy should be eliminated or consolidated into a single, concise description to improve readability and conciseness.**

**Response:** Thank you for your suggestion. We omitted those redundant lines.

**Line 76: I recommend that the authors add the following sentences in their introduction using the mentioned studies:
"The increase in multidrug-resistant (MDR) microorganisms triggering infections is growing worldwide and becoming more serious in developing countries."**

**Response:** Thank you. We have added this as suggested. See line: 68-69

**In line 76: It is important to use the update and so related reference according to the above comment:
Owaid HA, Al-Ouqaili MTS. Molecular and bacteriological investigations for the co-existence of CRISPR/Cas system and β-lactamases of types extended-spectrum and carbapenemases in Multidrug, extensive drug and Pandrug-Resistant Klebsiella pneumoniae. Saudi J Biol Sci. 2024 Jul;31(7):104022. doi: 10.1016/j.sjbs.2024.104022. Epub 2024 May 19. PMID: 38817398; PMCID: PMC11137337.
Rawaa A. Hussein, Shaymaa H. AL-Kubaisy, Mushtak T.S. Al-Ouqaili. The influence of efflux pump, outer membrane permeability and β-lactamase production on the resistance profile of multi, extensively and pandrug-resistant Klebsiella pneumoniae. Journal of Infection and Public Health, Volume 17, Issue 11, 2024.** [**https://doi.org/10.1016/j.jiph.2024.102544**](https://doi.org/10.1016/j.jiph.2024.102544)**.**

**Response:** Thank you for your recommendation. We appreciate it. We used one of the references mentioned to support our claim. However, we cannot use the other references, as they do not align with our claims. See reference 16.

**Lines 94–101: The manuscript provides detailed geographic coordinates and fruit types; however, the sampling design is not clearly defined. It remains unclear whether samples were collected using random, convenience, or systematic sampling. The authors should clarify the sampling strategy, inclusion and exclusion criteria, and whether the total sample size (n = 76) was statistically justified.**

**Response:** Thank you for your suggestion. We have now updated the manuscript and included the sampling method. See line: 99-100

**Lines 98–101: The number of samples per fruit type is highly uneven (e.g., apples n = 18 vs. dates n = 4). The authors should justify this disparity or discuss its potential impact on prevalence estimates, comparative analyses, and statistical power.**

**Response:** We acknowledge the uneven number of samples per fruit type (e.g., apples n = 18 vs. dates n = 4). This disparity was due to differences in fruit availability and prices, as well as funding limitations. Despite the variation, we aimed to ensure that each fruit type was represented proportionally to its availability in the sampled markets, and this limitation has been discussed in the manuscript regarding its potential impact on prevalence estimates, comparative analyses, and statistical power.

**Lines 105–108, 111: The enrichment step involves transferring 100 µL of PBS wash into 1 mL of nutrient broth; however, the rationale for this volume selection is not provided, nor is it clear whether selective enrichment media were considered. Additionally, the term “enriched culture” (line 111) appears to be a typographical error and should be corrected.**

**Response:** Thank you for your suggestion and concern. The 100 µL volume is selected to obtain a representative sample from the PBS wash while maintaining an appropriate inoculum size for bacterial enrichment in 1mL nutrient broth during overnight incubation. This method is well-established in our lab, and we use it frequently in our experiments. After the incubation, we transfer them to the selective media for E. faecalis isolation.

**Lines 154–160: The manuscript's claim that nine antibiotics correspond to eight groups requires clarification. Additionally, since rifampin (an ansamycin) is usually used as an adjuvant against Enterococcus spp., a justification for its inclusion in susceptibility testing should be provided.**

**Response:** We thank the reviewer for this comment. The classification has been clarified, and rifampin has been included in accordance with the CLSI 2024 guidelines.

**Lines 189–190, 197–198, 248: For prevalence across fruit types and biofilm formation categories, the authors report non-significant differences (p > 0.05) but do not provide test statistics or clarify which tests were used. Including the test type and exact p-values would enhance transparency.
Similarly, differences in antibiotic resistance among biofilm categories are described qualitatively, but statistical comparisons are lacking (Lines 242–248).**

**Response:** Thank you. We have now included the test name in the methods section of the manuscript. We have also noted that the p-value is non-significant at the end of the paragraph. However, these differences were not statistically significant (p>0.05).

**Lines 192–196, 205–208, 220–225: Biofilm formation was tested in only 18 out of 47 PCR-positive isolates (Lines 192–196), virulence genes in 12 isolates (Lines 205–208), and antibiotic resistance in 40 isolates (Lines 220–225). The rationale for selecting these subsets is not provided. The authors should justify the sample sizes for these analyses and discuss potential limitations regarding representativeness and statistical power.**

**Response:** Thank you for your concern. Due to funding and resource limitations, we couldn’t test all isolates. However, we have added this as one of our limitations in the end-of-discussions section.

**Lines 212–214, 231–239: Spearman and Pearson correlations between virulence genes and between antibiotics are reported, but the biological interpretation is not clearly explained. The authors should specify the rationale for these correlations and discuss potential confounding factors. Some correlations are repeated or redundant (Lines 235–237: Linezolid and Vancomycin are reported twice). The manuscript should remove duplicates to avoid confusion.**

**Response:** Thank you for the comment. We have revised the manuscript to include a clear biological interpretation of the Spearman and Pearson correlations in the Discussion section, explaining the rationale for analyzing co-occurrence patterns among virulence genes and antibiotic resistances, as well as potential confounding factors. Additionally, all duplicate correlations, including the repeated Linezolid–Vancomycin entry, have been removed to avoid confusion.

**Lines 250–253: The MDR prevalence and MAR index are reported, but methods for calculating MAR for each isolate are not linked to the results clearly. The authors should provide a table listing MAR values per isolate and clarify how multidrug resistance was defined (≥3 classes, consistent with Methods, Lines 160–162).**

**Response:** We thank the reviewer for this suggestion. The calculation of MDR (defined as resistance to ≥3 antibiotic classes, consistent with the Methods, Lines 164-165) and the MAR index for each isolate was already described in the Materials and Methods section under “Antibiotic Susceptibility Testing.” In Table 2, several isolates share identical resistance patterns and MAR values; to avoid an unnecessarily large table and to improve readability, these isolates were grouped, and the table was formatted accordingly. This approach preserves all relevant information while keeping the presentation concise and clear.

**Lines 261–269: The first paragraph repeats information about E. faecalis as a commensal organism and nosocomial pathogen, which was already described in the Introduction. Consider condensing these statements to focus on the relevance of your current study.**

**Response:** We appreciate your suggestion. We have revised this paragraph to remove repetitive background information about *E. faecalis* as a commensal and nosocomial pathogen. The text now focuses directly on the relevance and significance of our current study.

**Lines 270–288: While prevalence data are restated (Lines 271–273), the discussion relies heavily on speculation (e.g., poor hygiene, cross-contamination, dust) without citing study-specific observations. The authors should explicitly link environmental or vendor practices observed during sampling to the high prevalence findings.**

**Response:** Thank you for your concern. However, we have mentioned the specific studies at the end of the statement. The speculation we made was based on the following studies [reference 37-39] we cited in our manuscript.

Abat C, Huart M, Garcia V, Dubourg G, Raoult D. Enterococcus faecalis urinary-tract infections: Do they have a zoonotic origin? Journal of Infection. W.B. Saunders. 2016; 305–313.

Khanal AR, Timilsina RH, Sharma B, Pokharel B, Aryal R. Contaminated Water and an Indication of Risk: Examining Microbial Contamination in the Water Used by Consumers and Commercial Growers in Fresh Produce Systems in Nepal. J Food Prot. 2024;87.

Bartz FE, Lickness JS, Heredia N, de Aceituno AF, Newman KL, Hodge DW, et al. Contamination of fresh produce by microbial indicators on farms and in packing facilities: Elucidation of environmental routes. Appl Environ Microbiol. 2017;83.

**Line 293: It is preferred to use the following reference to support your updated citations: Al-Ouqaili, M.T.S., Al-Kubaisy, S.H.M., Al-Ani, N.F.I. Biofilm antimicrobial susceptibility pattern for selected antimicrobial agents against planktonic and sessile cells of clinical isolates of staphylococci using MICs, BICs and MBECs. Asian Journal of Pharmaceutics, volume 12, Issue 4, October-December 2018, Pages S1375-S1383.**
**Response:** Thank you for your suggestions. However, we added a citation, as described above.

**Line 358: The Conclusion should be objective, with further perspective, or should add at least a few sentences about future study/future perspective of it**

**Response:** Thank you for your suggestions. We have added a future perspective at the end of the conclusion.

“Future studies should focus on whole-genome sequencing to further investigate their transmission patterns in a public health context, and on a longitudinal study to identify risk factors that could help prevent the dissemination of AMR and pathogenicity.”

**Reviewer #4:**

Global comment

This manuscript investigates the presence of E. faecalis from fruit surfaces and characterizes the antibiotic resistance profile of these bacteria, detect its virulence genes and biofilm-forming ability, using selective media for bacterial identification and PCR assay. Phenotypic profile was obtained through the antibiotic susceptibility testing, and the genotypic profile was performed by PCR. Notably, the study highlights the frequency of multidrug-resistant (MDR) E. faecalis in fruits, a finding of global relevance due to the growing concern surrounding antimicrobial resistance.

The topic is timely and relevant, and the manuscript is scientifically supported. However, the language throughout the manuscript requires corrections for grammar, clarity, and style to ensure precision and readability. Moreover, some areas, require clarification and expansion to improve scientific rigor and reproducibility. Below are detailed comments and suggestions:

Comments

**Introduction**
**Line 49. The sentence was rewritten as follows: “Approximately 80 to 90 percent of human enterococcal infections are caused by E. faecalis, while most of the remaining cases are attributable to E. faecium.”**

**Response:** Thank you for your suggestion. We have updated the manuscript.

**Line 73. Rephrase. Multidrug resistance (MDR) typically refers to bacteria resistant to three or more distinct antibiotic classes.**

**Response:** Thank you for your suggestion. We have updated the manuscript.

**Line 76. Please check the entire manuscript. The names of genera, species, and genes should be written in italics.**
**Response:** Thank you for your suggestion. We have updated the manuscript accordingly.

**Material and Methods**

**Line 105. Please clarify how the fruits were processed. Were they washed before analysis, or were they immersed directly in PBS inside sterile bags?**

**Response:** Thank you. The fruits were brought in a zip-lock bag and washed with PBS in the lab. We mentioned that in line 103-107.

**Line 110. From bacterial growth in nutrient broth, please specify the volume or concentration used to inoculate the Enterococci agar base.**

**Response:** Thank you for your suggestion. We have now updated the manuscript.

**Line 156-160. The terms “access”, “watch”, and “reserve” are unclear in this context and make the procedure confusing. Please revise this section for clarity.**

**Response:** Thank you for your suggestions. We have now added a short meaning in the context of selecting antibiotics for treatment. Access- first line, commonly used, watch- higher resistance potential , and reserve- last resort antibiotic.

**Line 165. This information would be more appropriate in the section “Detection of Antibiotic Resistance and Virulence Genes”.**

**Response:** Thank you for your suggestion. We have updated the manuscript accordingly.

**Results**
**Line 180. Please rephrase. The entire manuscript requires revision for clarity and accuracy.**

**Response:** Thank you for your suggestion. We have now updated the manuscript.
**Line 186. It is incorrect to state that the PCR is positive. Instead, the isolates obtained from different fruits should be described as positive or negative for E. faecalis**.

**Response:** Thank you for your suggestion. We have now updated the manuscript.

**Line 193. Replace the term “samples” with “isolates”.**

**Response:** Thank you for your suggestion. We have now updated the manuscript.

**Figure 1, The text in the figure is not legible and should be improved.**

**Response:** Thank you for your suggestions. We have updated the figures with clear visibility.

**Line 200-202, the sentence should be moved to a table.**

**Response:** Thank you for the suggestion. The sentence in lines 200–202 has been revised and incorporated into the table legend to improve clarity and presentation.

**Line 205. Please explain why only 12 E. faecalis isolates were selected for further analysis.**

**Response:** Out of the 47 PCR-positive *E. faecalis* isolates, 12 were randomly selected for further analysis of virulence genes due to limitations in funding and availability of reagents.
**Figure 2. This figure does not present new data. Please add the total number of E. faecalis isolates. In addition, include information about the source of the isolates in Figures 2 and 3, and describe these data clearly in the Results section.**

**Response:** Thank you for your suggestions. We have updated the figures caption in the manuscript.

**Throughout the text, antibiotic names should not begin with a capital letter.**

**Response:** Thank you. We have updated the manuscript.

**Discussion**

**Line 261-269 This section repeats information already presented in the Introduction and should be revised.**

**Response:** We thank the reviewer for the comment. This section has been revised and condensed to avoid repetition of background information already presented in the Introduction.
**The Discussion should not be divided into subsections.**

**Response:** Thank you for your suggestion. We have updated the discussion.

**Line 281. According to a study, the prevalence 'of E. faecalis?’’**

**Response:** Thank you. We have now updated the manuscript.

“A recent study by Demisie et al. reported 50% of the prevalence in the fruit samples, which was considerably lower than that we found in the vegetable (86.5%) and salad (82.6%) samples.”

**Reviewer #5:**

Thank you for an interesting and potentially important manuscript reporting the detection of multidrug-resistant, biofilm-forming Enterococcus faecalis on fresh fruit surfaces, a first report for Bangladesh. The study has merit, but the authors should address several important methodological and interpretive issues before the manuscript can be finalized. My specific comments and suggestions are as follows-

**1. The manuscript uses different numbers of samples for different fruit types, but does not explain why sample sizes vary. This raises concerns about sampling bias and how representative the results are across fruit types. Add a clear explanation of the sampling design and justify the unequal sample sizes. If the unequal sizes were unavoidable (availability, seasonality, logistics), state this explicitly and discuss the limitation. If possible, include a short statistical justification**.

**Response:** We thank the reviewer for this observation. The number of samples per fruit type varied due to differences in availability during the sampling period, and samples were randomly selected to represent what was accessible at the time. We have clarified this in the manuscript and note that unequal sample sizes may limit representativeness across fruit types, while still providing preliminary insights into *E. faecalis* contamination in the region. We have also added as a limitation at the end of the discussions.

**2. The manuscript lists antibiotic susceptibility results but does not explain the rationale for selecting the particular antibiotic discs used. Provide a clear justification for each antibiotic tested: link each to its clinical relevance in human medicine and/or veterinary use in Bangladesh, and to commonly used classes for enterococci.**

**Response:** We thank the reviewer for this suggestion. The antibiotics tested were selected in accordance with the CLSI 2024 guidelines for *Enterococcus* species.
**3. The discussion should be revised to interpret resistance findings in the local epidemiological and antimicrobial-use (AMU) context in Bangladesh. Summarize which antibiotics are commonly prescribed/used in human clinical practice in Bangladesh for infections where enterococci are relevant (e.g., urinary tract infections, bloodstream infections) and which antibiotics are used in livestock/aquaculture/horticulture in the country.**

**Response:** We thank the reviewer for this suggestion. Antibiotic selection and interpretation were performed according to CLSI 2024 guidelines, and we have highlighted in the discussion that ampicillin is widely prescribed in Bangladesh for enterococcal infections.

**Reviewer #6:**

The manuscript addresses an important and underexplored public health issue and is well aligned with the One Health framework. It effectively integrates microbiological, molecular, and epidemiological approaches and provides novel regional data relevant to food-safety and antimicrobial-resistance (AMR) surveillance. Overall, the study has scientific merit and is potentially suitable for publication.
However, several revisions are required to strengthen methodological transparency, statistical rigor, and language quality. The key issues and recommended revisions are outlined below.

**Major Comments**

**1. Sampling Design and Representativeness**

**The overall sample size (n = 76) is relatively small and unevenly distributed across fruit types. In addition, the sampling strategy appears to be convenience-based and limited to a single city.**

**Recommendations:**

- Clearly acknowledge this as a limitation of the study.
- Avoid generalizing the findings to all fruit types or to the entire country of Bangladesh.
- Where appropriate, reframe conclusions to reflect the localized nature of the data.

**Response:** Thank you for your suggestions. We have now updated the manuscript accordingly. We have also acknowledged this as a limitation of the study.

**2. Subsampling for Biofilm Formation and Virulence Gene Analysis**

**Only 18 isolates were examined for biofilm-forming ability, and 12 isolates for virulence gene detection, without sufficient justification for this subsampling approach.**

**Recommendations:**

- Provide a clear rationale for the random selection of isolates (e.g., logistical or resource constraints).
- Explicitly state that the reported prevalence of biofilm formation and virulence genes may be underestimated.
- If feasible, expand the number of isolates tested or provide a statistical justification for the chosen subsample size.

**Response:** We thank the reviewer for this suggestion. We have provided a rationale for the random selection of isolates as a limitation at the end of the discussion. Due to funding limitations, we were not able to expand the subsample size.

**3. Interpretation of Antibiotic Resistance Findings**

**The reported high resistance rates to vancomycin and linezolid are concerning and atypical for food-derived Enterococcus isolates.**

**Recommendations:**
• Provide a more detailed discussion of potential methodological limitations, including the use of disc diffusion for glycopeptides and oxazolidinones.

• Discuss possible environmental, anthropogenic, or ecological drivers that could explain these findings.

• Cite relevant CLSI cautions and limitations regarding susceptibility testing for these antibiotic classes.

**Response:** We thank the reviewer for this important observation. Antimicrobial susceptibility testing was performed according to CLSI 2024; however, we acknowledge that disc diffusion for vancomycin and linezolid has recognized limitations, and MIC-based methods- recommended by CLSI for greater accuracy could not be performed due to resource constraints. We have also updated the ecological drivers (environmental exposure or horizontal gene transfer) for resistance to vancomycin and linezolid.

**4. Statistical Analysis**

Some of the reported correlations (e.g., Linezolid–Vancomycin, ρ = 1.000) suggest potential artefacts related to small sample sizes. In addition, no correction for multiple testing is mentioned.

**Recommendations:**
• Acknowledge the exploratory nature of the correlation analyses.

• Include a statement addressing the increased risk of type I error due to multiple comparisons.

• Interpret correlation results with appropriate caution.

**Response:** Thank you for your valuable suggestions. We have added a statement in both the results and the discussion sections to interpret this correlation result with appropriate caution.

**5. Language and Editorial Quality**

**The manuscript contains repetitive statements, overly long sentences, minor grammatical errors, and occasional inconsistencies in terminology (e.g., use of “weak” versus “intermediate” biofilm categories).
Recommendation:**
• Professional English language editing is strongly recommended prior to resubmission to improve clarity, consistency, and readability.

**Response:** Thank you for your suggestions. We have tried to improve the clarity in the whole manuscript.

**Section-by-Section Comments**

**Abstract**
The abstract is well structured and data-rich. However, it would benefit from:

• Reducing excessive numerical detail.

• More clearly highlighting the key findings and their policy or public health relevance.

**Response:** Thank you for your suggestions. We have reduced the numerical value and highlighted the key findings in the abstract.

**Introduction**
The introduction is scientifically sound and well referenced. Improvements are recommended to:

• Reduce redundancy, particularly regarding antimicrobial resistance mechanisms.

• Improve conciseness and narrative flow.

**Response:** Thank you for your suggestions. We have reduced the redundancy and improved the introduction.

**Materials and Methods**

This section is generally robust. The ethics statement marked as “N/A” is acceptable; however:

- The authors should explicitly clarify that the study did not involve human or animal subjects.
- Additional justification should be provided for partial molecular testing of isolates.

**Response:** Thank you for your suggestions. We have updated the manuscript and mentioned the ethics statement. Due to funding limitations, we had to do partial molecular testing of isolates.

**Results**
The results are clearly presented; however:

- Tables require harmonization to ensure consistency in numbering and formatting.
- Figure captions should be expanded to be fully self-explanatory.

**Response:** Thank you for your suggestions. We have updated this in the manuscript.

**Discussion**
The discussion is well contextualized within the existing literature. To further strengthen this section, the authors should:

• Avoid speculation beyond what is directly supported by the data.

• Strengthen comparisons with studies from South Asia and similar settings.

• Include a clearly defined Limitations subsection.

**Response:** We thank the reviewer for the positive feedback and constructive suggestions. We have carefully revised the Discussion to ensure that interpretations are fully supported by our data and to strengthen comparisons with studies from South Asia and similar settings. Additionally, we have added a clearly defined Limitations subsection to highlight the study’s scope and constraints.

**Conclusions**
The conclusions are appropriate and supported by the data. The section could be further strengthened by:
• Adding a brief statement on policy or regulatory implications, such as market hygiene practices, food safety monitoring, or routine AMR surveillance.

**Response:** Thank you for your comments and suggestions. We have updated the manuscript.
