# Supplementary material for: Fresh fruit surface: A potential source for multidrug-resistant biofilm-forming Enterococcus faecalis having potential public health significance
Source: PLoS One. 2026 Aug 5;21(8):e0355410. doi: 10.1371/journal.pone.0355410 (PMC13440884; doi:10.1371/journal.pone.0355410)
Supplement: Supplementary file 8 [file pone.0355410.s008.docx]

6. Review Comments to the Author

Please use the space provided to explain your answers to the questions above. You may also include additional comments for the author, including concerns about dual publication, research ethics, or publication ethics. (Please upload your review as an attachment if it exceeds 20,000 characters)

Reviewer #6: Thank you for your careful revision. The manuscript has improved substantially in clarity, organization, and transparency. The study is relevant and contributes useful baseline data on antimicrobial-resistant Enterococcus faecalis in fresh fruits.

However, the following issues remain that need to be addressed

**1. The study confirms 47 isolates of E. faecalis, yet:**

• Biofilm analysis: 18 isolates

• Virulence genes: 12 isolates

This represents a significant reduction in analytical coverage.

While you have acknowledged this limitation, the manuscript still states that virulence genes and biofilm traits are “widely distributed”, which is not fully supported by the subsampled data.

To address this the author should:

• Rephrase conclusions to indicate findings are preliminary or indicative

• Avoid generalizing to all isolates

• Explicitly state that prevalence may be over- or under-estimated

**Response:** Thank you so much for your suggestions. We have rephrased our claims and updated the manuscript. See line: 386-388

**2. The reported resistance levels: Vancomycin: 47.5% and Linezolid: 47.5% are unexpectedly high for food-derived isolates and require stronger caution. Although you mention CLSI limitations, this is not sufficiently emphasized.**

The Critical issue here is that: Disk diffusion is not the preferred method for these antibiotics in enterococci, and may lead to inaccurate classification.

To address this the author is Required to:

• Add a strong statement that:

o These results may overestimate resistance

o MIC-based confirmation is required

• Avoid implying confirmed presence of:

o Vancomycin-resistant enterococci (VRE)

o Linezolid-resistant strains

• Tone down conclusions accordingly, especially in the Conclusion section

**Response:** Thank you for your suggestions. Now, we have modified and rephrased the statements to tone them down. See line: 349-350

**3. The manuscript reports correlation values (e.g., ρ = 1.000) that are statistically questionable, especially given small sample sizes. The concerns here are:**

• Likely artefacts of small sample size

• No correction for multiple comparisons

• Limited biological interpretability

The required revisions in this circumstances is for the author to:

• Clearly state that:

o Correlation analysis is exploratory

o Results are hypothesis-generating only

• Add a statement on risk of Type I error

• Interpret correlations cautiously or move to supplementary material

**Response:** Thank you for your feedback. We have updated the manuscript. See line: 365-370

4. The study includes: 76 fruit samples from a single city (Mymensingh), Uneven distribution across fruit types, This limits generalizability.

The author should:

• Clearly describe sampling as:

o “localized” or “site-specific”

• Emphasize findings as:

o baseline data, not national estimates

• Avoid general statements about broader populations

**Response:** Thank you. We have updated the manuscript. See line: 399

5. The manuscript suggests that fruit surfaces pose a “significant risk of transmission to humans”

This statement is too strong given that there is:

• No exposure assessment

• No transmission pathway analysis

• No genomic linkage to human infections

Required revisions:

• Replace with cautious phrasing such as:

o “may pose a potential risk”

o “could contribute to exposure”

• Avoid causal or definitive public health claims

**Response:** Thank you for your suggestions. Now we have rephrased the statement. See line: 406

6. The following minor improvements in language and presentation are also recommended:

a. The manuscript Language and Grammar has improved but still contains

o Typographical errors

o Awkward phrasing

Professional language editing is recommended

b. MAR index formula is included but should be clearly linked to results

c. Clarify how isolates were selected for subsampling (randomization process)

d. CRA is correctly described

e. However, emphasize more clearly that it is:

o qualitative

o less precise than quantitative assays

**Response:** Thank you for your suggestions. We have updated the manuscript.

f. Ensure all figures and tables:

o Include sample sizes (n)

o Have self-explanatory captions

g. Ensure consistency in formatting

**Response:** Thank you for your suggestions. We have updated the manuscript.
