# Supplementary material for: Fresh fruit surface: A potential source for multidrug-resistant biofilm-forming Enterococcus faecalis having potential public health significance
Source: PLoS One. 2026 Aug 5;21(8):e0355410. doi: 10.1371/journal.pone.0355410 (PMC13440884; doi:10.1371/journal.pone.0355410)
Supplement: Peer review history

# Peer review: Decision Letter 0

**DOI:** 10.1371/journal.pone.0355410.r001

**Authors:** Leonard Ighodalo Uzairue

22 Feb 2026

PONE-D-25-60195

Fresh fruit surface: a potential source for multidrug-resistant biofilm-forming Enterococcus faecalis having potential public health significance

PLOS One

Dear Dr. Rahman,

Thank you for submitting your manuscript to PLOS ONE. After careful consideration, we feel that it has merit but does not fully meet PLOS ONE’s publication criteria as it currently stands. Therefore, we invite you to submit a revised version of the manuscript that addresses the points raised during the review process.

Please submit your revised manuscript by Apr 08 2026 11:59PM. If you will need more time than this to complete your revisions, please reply to this message or contact the journal office at plosone@plos.org. When you're ready to submit your revision, log on to https://www.editorialmanager.com/pone/ and select the 'Submissions Needing Revision' folder to locate your manuscript file.

Please include the following items when submitting your revised manuscript:

- A letter that responds to each point raised by the academic editor and reviewer(s). You should upload this letter as a separate file labeled 'Response to Reviewers'.
- A marked-up copy of your manuscript that highlights changes made to the original version. You should upload this as a separate file labeled 'Revised Manuscript with Track Changes'.
- An unmarked version of your revised paper without tracked changes. You should upload this as a separate file labeled 'Manuscript'.

If you would like to make changes to your financial disclosure, please include your updated statement in your cover letter. Guidelines for resubmitting your figure files are available below the reviewer comments at the end of this letter.

If applicable, we recommend that you deposit your laboratory protocols in protocols.io to enhance the reproducibility of your results. Protocols.io assigns your protocol its own identifier (DOI) so that it can be cited independently in the future. For instructions see: https://journals.plos.org/plosone/s/submission-guidelines#loc-laboratory-protocols. Additionally, PLOS ONE offers an option for publishing peer-reviewed Lab Protocol articles, which describe protocols hosted on protocols.io. Read more information on sharing protocols at https://plos.org/protocols?utm_medium=editorial-email&utm_source=authorletters&utm_campaign=protocols.

We look forward to receiving your revised manuscript.

Kind regards,

Leonard Ighodalo Uzairue, PhD

Academic Editor

PLOS One

Journal Requirements:

When submitting your revision, we need you to address these additional requirements.

1. Please ensure that your manuscript meets PLOS ONE's style requirements, including those for file naming. The PLOS ONE style templates can be found at

https://journals.plos.org/plosone/s/file?id=wjVg/PLOSOne_formatting_sample_main_body.pdf and https://journals.plos.org/plosone/s/file?id=ba62/PLOSOne_formatting_sample_title_authors_affiliations.pdf

2. Please include a complete copy of PLOS’ questionnaire on inclusivity in global research in your revised manuscript. Our policy for research in this area aims to improve transparency in the reporting of research performed outside of researchers’ own country or community. The policy applies to researchers who have travelled to a different country to conduct research, research with Indigenous populations or their lands, and research on cultural artefacts. The questionnaire can also be requested at the journal’s discretion for any other submissions, even if these conditions are not met.  Please find more information on the policy and a link to download a blank copy of the questionnaire here: https://journals.plos.org/plosone/s/best-practices-in-research-reporting. Please upload a completed version of your questionnaire as Supporting Information when you resubmit your manuscript.

3. We noticed you have some minor occurrence of overlapping text with the following previous publication(s), which needs to be addressed:

https://pubmed.ncbi.nlm.nih.gov/40388390/

In your revision ensure you cite all your sources (including your own works), and quote or rephrase any duplicated text outside the methods section. Further consideration is dependent on these concerns being addressed.

4. When completing the data availability statement of the submission form, you indicated that you will make your data available on acceptance. We strongly recommend all authors decide on a data sharing plan before acceptance, as the process can be lengthy and hold up publication timelines. Please note that, though access restrictions are acceptable now, your entire data will need to be made freely accessible if your manuscript is accepted for publication. This policy applies to all data except where public deposition would breach compliance with the protocol approved by your research ethics board. If you are unable to adhere to our open data policy, please kindly revise your statement to explain your reasoning and we will seek the editor's input on an exemption. Please be assured that, once you have provided your new statement, the assessment of your exemption will not hold up the peer review process.

5. Please include a copy of Table 2 which you refer to in your text on page 10, 12.

6. If the reviewer comments include a recommendation to cite specific previously published works, please review and evaluate these publications to determine whether they are relevant and should be cited. There is no requirement to cite these works unless the editor has indicated otherwise.

Additional Editor Comments :

Thank you for your manuscript; kindly take note of the following point.

1. A reviewer has proposed additional articles for possible inclusion in the manuscript. It is essential to carefully review each recommendation and determine whether it aligns with the scope of the section where these references are to be added. If any suggested articles are found to be outside the defined scope, please provide a clear explanation and justification for their exclusion. Articles that do not fit within the intended context should not be incorporated into the manuscript.

2. The current presentation of the information on lines 231 to 239 lacks a supporting table. Please organise this content into a table to enhance clarity and ease of interpretation. Additionally, ensure that the statistical methods section explicitly describes the process used to calculate and interpret the correlations, thus promoting reproducibility and transparency in the analysis.

[Note: HTML markup is below. Please do not edit.]

Reviewers' comments:

Reviewer's Responses to Questions

**Comments to the Author**

1. Is the manuscript technically sound, and do the data support the conclusions?

The manuscript must describe a technically sound piece of scientific research with data that supports the conclusions. Experiments must have been conducted rigorously, with appropriate controls, replication, and sample sizes. The conclusions must be drawn appropriately based on the data presented.

Reviewer #1: Yes

Reviewer #2: Yes

Reviewer #3: Yes

Reviewer #4: Yes

Reviewer #5: Yes

Reviewer #6: Yes

**********

2. Has the statistical analysis been performed appropriately and rigorously?

Reviewer #1: Yes

Reviewer #2: Yes

Reviewer #3: Yes

Reviewer #4: Yes

Reviewer #5: Yes

Reviewer #6: Yes

**********

3. Have the authors made all data underlying the findings in their manuscript fully available?

The PLOS Data policy requires authors to make all data underlying the findings described in their manuscript fully available without restriction, with rare exception (please refer to the Data Availability Statement in the manuscript PDF file). The data should be provided as part of the manuscript or its supporting information, or deposited to a public repository. For example, in addition to summary statistics, the data points behind means, medians and variance measures should be available. If there are restrictions on publicly sharing data—e.g. participant privacy or use of data from a third party—those must be specified.

Reviewer #1: Yes

Reviewer #2: Yes

Reviewer #3: Yes

Reviewer #4: Yes

Reviewer #5: Yes

Reviewer #6: Yes

**********

4. Is the manuscript presented in an intelligible fashion and written in standard English?

PLOS ONE does not copyedit accepted manuscripts, so the language in submitted articles must be clear, correct, and unambiguous. Any typographical or grammatical errors should be corrected at revision, so please note any specific errors here.

Reviewer #1: Yes

Reviewer #2: Yes

Reviewer #3: Yes

Reviewer #4: Yes

Reviewer #5: Yes

Reviewer #6: Yes

**********

5. Review Comments to the Author

Please use the space provided to explain your answers to the questions above. You may also include additional comments for the author, including concerns about dual publication, research ethics, or publication ethics. (Please upload your review as an attachment if it exceeds 20,000 characters)

Reviewer #1: The manuscript described molecular detection and characterization of MDR Enterococcus faecalis associated with fresh fruit surface sold in Mymensingh area. In addition to AMR, biofilm formation was also observed in this study. Considering it as the first such study in Bangladesh and the public health importance of biofilm forming MDR E. faecalis the study carries significant and crucial message for the food sellers/vendors, consumers and policy makers.

Specific Comments.

1. How much DNA was used in each PCR as template?

2. Congo Red assay was used for biofilm detection. Describe the principle/mechanism of Cogo Red test.

3. Several virulence genes were used, please mention the functions of these genes, many are not mentioned in the manuscript.

4. What was the rationale of selecting those 9 antibiotics in the AST?

5. Please remove the CI from the values e.g, in line 180 instead of 47 (61.84%, CI95: 50.60–71.94), simply write 47 (61.84%)

6. There are different types of fruits available in the market. Why were these particular fruits were selected?

7. Line 2412, Moreover, Strong biofilm-forming E. faecalis isolates exhibited higher resistance to certain antibiotics compared to intermediate and non-biofilm formers, in discussion explain this observed phenomenon.

8. Line 252, Furthermore, all the patterns showed resistance to a similar number of antibiotics and classes. any explanation why similar patterns were observed??

Reviewer #2: The manuscript entitlted ‘’ Fresh fruit surface: a potential source for multidrug-resistant biofilm-forming Enterococcus faecalis having potential public health significance’’ presented a well-designed and timely investigation into the occurrence, AMR, biofilm formation and virulence gene profiles of E. faecalis from fresh fruit surfaces in Bangladesh. The study addressed an important gap in food safety and One Health research, particularly in low- and middle income country where surveillance data on antimicrobial-resistant bacteria in fresh produce remain scarce.

The methodology is clearly described, results are logically structured, and discussion is well contextualized within the existing literature. Overall, the manuscript makes a valuable contribution to understanding the public health implication of antimicrobial-resistant enterococci in the food chain.

Besides its novelty and strength, several limitation should be acknowledged more explicitly to make it scientifically sound.

Limitations and Suggesstions

1. This study analyzed 76 fruit samples of different types exclusively from Mymensingh city, which is not adequate for an exploratory investigation, and restricts generalizability. Moreover, seasonal variation was not explicitly assessed. Thus, results should be interpred as region-specific baseline data rather than national prevalence estimates

2. Only 18 isolates were tested for biofilm formation and 12 isolates for virulence gene profiling, despite 47 PCR-confirmed isolates. The subsampling approach is acceptable for preliminary characterization but limits inferential depth. The authors are suggested to examine all the 47 isolates to make the result scientifically robust.

3. Biofilm formation was assessed using only Congo Red Agar (CRA) method, which is qualitative and phenotype-dependent. CRA provides rapid screening but may underestimate or overestimate true biofilm biomass.

4. Among resistance genes only blaTEM was screened. Key enterococcal resistance genes (e.g. vanA, vanB, ermB tetM, optrA, postA) were not investigated, despite high phenotypic resistance to vancomycin and linezolid. Phenotype-genotype discordance cannot be fully resolved without broader resistance gene profiling.

5. The study relies on PCR-based detection rather than whole-genome sequencing. Consequently, clonal relationship, plasmid content, mobile genetic elements, and resistome structure remain unexplored. WGS in recommended for future studies to confirm transmission dynamics and One Health linkages.

Reviewer #3: Dear Editor,

This manuscript introduces a unique concept in an area that is both intriguing and important for medical research. The topic is timely and is likely to engage the journal's readers. Additionally, the manuscript is well-written, presenting ideas clearly and logically.

Dear Authors,

You are doing excellent work, and this manuscript reflects a great deal of effort and thoughtful research. The study is promising and contributes meaningfully to the field. However, there are a few important points that require further clarification and revision to enhance the overall quality and impact of the manuscript.

Line 44: The Introduction covers several important themes (enterococci biology, public health relevance, fruit contamination, and antibiotic resistance). However, the narrative would benefit from improved coherence and a clearer logical flow. The authors are encouraged to better integrate these topics, explicitly linking Enterococcus faecalis ecology with fruit-associated transmission and public health implications.

Line 46: It is preferred to use the following reference to support your updated citations: Alduhaidhawi AHM, AlHuchaimi SN, Al-Mayah TA, Al-Ouqaili MTS, Alkafaas SS, Muthupandian S, Saki M. Prevalence of CRISPR-Cas Systems and Their Possible Association with Antibiotic Resistance in Enterococcus faecalis and Enterococcus faecium Collected from Hospital Wastewater. Infect Drug Resist. 2022 Mar 19;15:1143-1154. Doi: 10.2147/IDR.S358248. PMID: 35340673; PMCID: PMC8942119.

Statements suggesting that consumption of fresh fruits may increase the “baseline level of antibiotic resistance” in humans (lines 69–71) are potentially overstated. These claims should be more carefully qualified and supported with stronger evidence or rephrased to avoid causal implications not fully substantiated by current data.

Lines 76–79 repeat nearly identical statements regarding the intrinsic resistance mechanisms of E. faecalis. This redundancy should be eliminated or consolidated into a single, concise description to improve readability and conciseness.

Line 76: I recommend that the authors add the following sentences in their introduction using the mentioned studies:

"The increase in multidrug-resistant (MDR) microorganisms triggering infections is growing worldwide and becoming more serious in developing countries."

In line 76: It is important to use the update and so related reference according to the above comment:

Owaid HA, Al-Ouqaili MTS. Molecular and bacteriological investigations for the co-existence of CRISPR/Cas system and β-lactamases of types extended-spectrum and carbapenemases in Multidrug, extensive drug and Pandrug-Resistant Klebsiella pneumoniae. Saudi J Biol Sci. 2024 Jul;31(7):104022. doi: 10.1016/j.sjbs.2024.104022. Epub 2024 May 19. PMID: 38817398; PMCID: PMC11137337.

Rawaa A. Hussein, Shaymaa H. AL-Kubaisy, Mushtak T.S. Al-Ouqaili. The influence of efflux pump, outer membrane permeability and β-lactamase production on the resistance profile of multi, extensively and pandrug-resistant Klebsiella pneumoniae. Journal of Infection and Public Health, Volume 17, Issue 11, 2024. https://doi.org/10.1016/j.jiph.2024.102544.

Lines 94–101: The manuscript provides detailed geographic coordinates and fruit types; however, the sampling design is not clearly defined. It remains unclear whether samples were collected using random, convenience, or systematic sampling. The authors should clarify the sampling strategy, inclusion and exclusion criteria, and whether the total sample size (n = 76) was statistically justified.

Lines 98–101:The number of samples per fruit type is highly uneven (e.g., apples n = 18 vs. dates n = 4). The authors should justify this disparity or discuss its potential impact on prevalence estimates, comparative analyses, and statistical power.

Lines 105–108, 111: The enrichment step involves transferring 100 µL of PBS wash into 1 mL of nutrient broth; however, the rationale for this volume selection is not provided, nor is it clear whether selective enrichment media were considered. Additionally, the term “enriched culture” (line 111) appears to be a typographical error and should be corrected.

Lines 154–160: The manuscript's claim that nine antibiotics correspond to eight groups requires clarification. Additionally, since rifampin (an ansamycin) is usually used as an adjuvant against Enterococcus spp., a justification for its inclusion in susceptibility testing should be provided.

Lines 189–190, 197–198, 248: For prevalence across fruit types and biofilm formation categories, the authors report non-significant differences (p > 0.05) but do not provide test statistics or clarify which tests were used. Including the test type and exact p-values would enhance transparency.

Similarly, differences in antibiotic resistance among biofilm categories are described qualitatively, but statistical comparisons are lacking (Lines 242–248).

Lines 192–196, 205–208, 220–225: Biofilm formation was tested in only 18 out of 47 PCR-positive isolates (Lines 192–196), virulence genes in 12 isolates (Lines 205–208), and antibiotic resistance in 40 isolates (Lines 220–225). The rationale for selecting these subsets is not provided. The authors should justify the sample sizes for these analyses and discuss potential limitations regarding representativeness and statistical power.

Lines 212–214, 231–239: Spearman and Pearson correlations between virulence genes and between antibiotics are reported, but the biological interpretation is not clearly explained. The authors should specify the rationale for these correlations and discuss potential confounding factors.

Some correlations are repeated or redundant (Lines 235–237: Linezolid and Vancomycin are reported twice). The manuscript should remove duplicates to avoid confusion.

Lines 250–253: The MDR prevalence and MAR index are reported, but methods for calculating MAR for each isolate are not linked to the results clearly. The authors should provide a table listing MAR values per isolate and clarify how multidrug resistance was defined (≥3 classes, consistent with Methods, Lines 160–162).

Lines 261–269: The first paragraph repeats information about E. faecalis as a commensal organism and nosocomial pathogen, which was already described in the Introduction. Consider condensing these statements to focus on the relevance of your current study.

Lines 270–288: While prevalence data are restated (Lines 271–273), the discussion relies heavily on speculation (e.g., poor hygiene, cross-contamination, dust) without citing study-specific observations. The authors should explicitly link environmental or vendor practices observed during sampling to the high prevalence findings.

Line 293: It is preferred to use the following reference to support your updated citations: Al-Ouqaili, M.T.S., Al-Kubaisy, S.H.M., Al-Ani, N.F.I. Biofilm antimicrobial susceptibility pattern for selected antimicrobial agents against planktonic and sessile cells of clinical isolates of staphylococci using MICs, BICs and MBECs. Asian Journal of Pharmaceutics, volume 12, Issue 4, October-December 2018, Pages S1375-S1383.

Line 358: The Conclusion should be objective, with further perspective, or should add at least a few sentences about future study/future perspective of it

Reviewer #4: Global comment

This manuscript investigates the presence of E. faecalis from fruit surfaces and characterizes the antibiotic resistance profile of these bacteria, detect its virulence genes and biofilm-forming ability, using selective media for bacterial identification and PCR assay. Phenotypic profile was obtained through the antibiotic susceptibility testing, and the genotypic profile was performed by PCR. Notably, the study highlights the frequency of multidrug-resistant (MDR) E. faecalis in fruits, a finding of global relevance due to the growing concern surrounding antimicrobial resistance.

The topic is timely and relevant, and the manuscript is scientifically supported. However, the language throughout the manuscript requires corrections for grammar, clarity, and style to ensure precision and readability. Moreover, some areas, require clarification and expansion to improve scientific rigor and reproducibility. Below are detailed comments and suggestions:

Comments

Introduction

Line 49. The sentence was rewritten as follows: “Approximately 80 to 90 percent of human enterococcal infections are caused by E. faecalis, while most of the remaining cases are attributable to E. faecium.”

Line 73. Rephrase. Multidrug resistance (MDR) typically refers to bacteria resistant to three or more distinct antibiotic classes.

Line 76. Please check the entire manuscript. The names of genera, species, and genes should be written in italics.

Material and Methods

Line 105. Please clarify how the fruits were processed. Were they washed before analysis, or were they immersed directly in PBS inside sterile bags?

Line 110. From bacterial growth in nutrient broth, please specify the volume or concentration used to inoculate the Enterococcosel agar base.

Line 156-160. The terms “access”, “watch”, and “reserve” are unclear in this context and make the procedure confusing. Please revise this section for clarity.

Line 165. This information would be more appropriate in the section “Detection of Antibiotic Resistance and Virulence Genes”.

Results

Line 180. Please rephrase. The entire manuscript requires revision for clarity and accuracy.

Line 186. It is incorrect to state that the PCR is positive. Instead, the isolates obtained from different fruits should be described as positive or negative for E. faecalis.

Line 193. Replace the term “samples” with “isolates”.

Figure 1, The text in the figure is not legible and should be improved.

Line 200-202, the sentence should be moved to a table.

Line 205. Please explain why only 12 E. faecalis isolates were selected for further analysis.

Figure 2. This figure does not present new data. Please add the total number of E. faecalis isolates. In addition, include information about the source of the isolates in Figures 2 and 3, and describe these data clearly in the Results section.

Throughout the text, the names of antibiotics should not begin with a capital letter.

Discussion

Line 261-269 This section repeats information already presented in the Introduction and should be revised.

The Discussion should not be divided into subsections.

Line 281. According to a study, the prevalence 'of E. faecalis?’’

Reviewer #5: Thank you for an interesting and potentially important manuscript reporting the detection of multidrug-resistant, biofilm-forming Enterococcus faecalis on fresh fruit surfaces, a first report for Bangladesh. The study has merit, but the authors should address several important methodological and interpretive issues before the manuscript can be finalized. My specific comments and suggestions are as follows-

1. The manuscript uses different numbers of samples for different fruit types, but does not explain why sample sizes vary. This raises concerns about sampling bias and how representative the results are across fruit types. Add a clear explanation of the sampling design and justify the unequal sample sizes. If the unequal sizes were unavoidable (availability, seasonality, logistics), state this explicitly and discuss the limitation. If possible, include a short statistical justification.

2. The manuscript lists antibiotic susceptibility results but does not explain the rationale for selecting the particular antibiotic discs used. Provide a clear justification for each antibiotic tested: link each to its clinical relevance in human medicine and/or veterinary use in Bangladesh, and to commonly used classes for enterococci.

3. The discussion should be revised to interpret resistance findings in the local epidemiological and antimicrobial-use (AMU) context in Bangladesh. Summarize which antibiotics are commonly prescribed/used in human clinical practice in Bangladesh for infections where enterococci are relevant (e.g., urinary tract infections, bloodstream infections) and which antibiotics are used in livestock/aquaculture/horticulture in the country.

Reviewer #6: The manuscript addresses an important and underexplored public health issue and is well aligned with the One Health framework. It effectively integrates microbiological, molecular, and epidemiological approaches and provides novel regional data relevant to food safety and antimicrobial resistance (AMR) surveillance. Overall, the study has scientific merit and is potentially suitable for publication.

However, several revisions are required to strengthen methodological transparency, statistical rigor, and language quality. The key issues and recommended revisions are outlined below.

Major Comments

1. Sampling Design and Representativeness

The overall sample size (n = 76) is relatively small and unevenly distributed across fruit types. In addition, the sampling strategy appears to be convenience-based and limited to a single city.

Recommendations:

• Clearly acknowledge this as a limitation of the study.

• Avoid generalizing the findings to all fruit types or to the entire country of Bangladesh.

• Where appropriate, reframe conclusions to reflect the localized nature of the data.

2. Subsampling for Biofilm Formation and Virulence Gene Analysis

Only 18 isolates were examined for biofilm-forming ability and 12 isolates for virulence gene detection, without sufficient justification for this subsampling approach.

Recommendations:

• Provide a clear rationale for the random selection of isolates (e.g., logistical or resource constraints).

• Explicitly state that the reported prevalence of biofilm formation and virulence genes may be underestimated.

• If feasible, expand the number of isolates tested or provide a statistical justification for the chosen subsample size.

3. Interpretation of Antibiotic Resistance Findings

The reported high resistance rates to vancomycin and linezolid are concerning and atypical for food-derived Enterococcus isolates.

Recommendations:

• Provide a more detailed discussion of potential methodological limitations, including the use of disc diffusion for glycopeptides and oxazolidinones.

• Discuss possible environmental, anthropogenic, or ecological drivers that could explain these findings.

• Cite relevant CLSI cautions and limitations regarding susceptibility testing for these antibiotic classes.

4. Statistical Analysis

Some of the reported correlations (e.g., Linezolid–Vancomycin, ρ = 1.000) suggest potential artefacts related to small sample sizes. In addition, no correction for multiple testing is mentioned.

Recommendations:

• Acknowledge the exploratory nature of the correlation analyses.

• Include a statement addressing the increased risk of type I error due to multiple comparisons.

• Interpret correlation results with appropriate caution.

5. Language and Editorial Quality

The manuscript contains repetitive statements, overly long sentences, minor grammatical errors, and occasional inconsistencies in terminology (e.g., use of “weak” versus “intermediate” biofilm categories).

Recommendation:

• Professional English language editing is strongly recommended prior to resubmission to improve clarity, consistency, and readability.

Section-by-Section Comments

Abstract

The abstract is well structured and data-rich. However, it would benefit from:

• Reducing excessive numerical detail.

• More clearly highlighting the key findings and their policy or public health relevance.

Introduction

The introduction is scientifically sound and well referenced. Improvements are recommended to:

• Reduce redundancy, particularly regarding antimicrobial resistance mechanisms.

• Improve conciseness and narrative flow.

Materials and Methods

This section is generally robust. The ethics statement marked as “N/A” is acceptable; however:

• The authors should explicitly clarify that the study did not involve human or animal subjects.

• Additional justification should be provided for partial molecular testing of isolates.

Results

The results are clearly presented; however:

• Tables require harmonization to ensure consistency in numbering and formatting.

• Figure captions should be expanded to be fully self-explanatory.

Discussion

The discussion is well contextualized within the existing literature. To further strengthen this section, the authors should:

• Avoid speculation beyond what is directly supported by the data.

• Strengthen comparisons with studies from South Asia and similar settings.

• Include a clearly defined Limitations subsection.

Conclusions

The conclusions are appropriate and supported by the data. The section could be further strengthened by:

• Adding a brief statement on policy or regulatory implications, such as market hygiene practices, food safety monitoring, or routine AMR surveillance.

**********

6. PLOS authors have the option to publish the peer review history of their article (what does this mean?). If published, this will include your full peer review and any attached files.

If you choose “no”, your identity will remain anonymous but your review may still be made public.

**Do you want your identity to be public for this peer review?**  For information about this choice, including consent withdrawal, please see our Privacy Policy.

Reviewer #1: No

Reviewer #2: No

Reviewer #3: No

Reviewer #4: No

Reviewer #5: **Yes:**  Sukumar Saha

Reviewer #6: **Yes:**  Lawan Adamu

**********

[NOTE: If reviewer comments were submitted as an attachment file, they will be attached to this email and accessible via the submission site. Please log into your account, locate the manuscript record, and check for the action link "View Attachments". If this link does not appear, there are no attachment files.]

To ensure your figures meet our technical requirements, please review our figure guidelines: https://journals.plos.org/plosone/s/figures

You may also use PLOS’s free figure tool, NAAS, to help you prepare publication quality figures: https://journals.plos.org/plosone/s/figures#loc-tools-for-figure-preparation.

NAAS will assess whether your figures meet our technical requirements by comparing each figure against our figure specifications.

Attachment Submitted filename: Reviewer comments-PONE-D-25-60195_reviewer.pdf

# Author comment: Author response to Decision Letter 1

**DOI:** 10.1371/journal.pone.0355410.r002

15 Apr 2026

Reviewer #1: The manuscript described molecular detection and characterization of MDR Enterococcus faecalis associated with fresh fruit surface sold in Mymensingh area. In addition to AMR, biofilm formation was also observed in this study. Considering it as the first such study in Bangladesh and the public health importance of biofilm forming MDR E. faecalis the study carries significant and crucial message for the food sellers/vendors, consumers and policy makers.

Specific Comment:

1. How much DNA was used in each PCR as template?

Response: Thank you. In line 128, it is mentioned that we used 50 to 60 ng of DNA as template.

2. Congo Red assay was used for biofilm detection. Describe the principle/mechanism of Cogo Red test.

Response: Thank you. Now, we have updated the manuscript with adequate information from line 139 to line 142.

3. Several virulence genes were used. Please mention the functions of these genes; many are not mentioned in the manuscript.

Response: Thank you. We have updated the gene functions in line 149 to line 150.

4. What was the rationale for selecting those 9 antibiotics in the AST?

Response: Thank you. We have mentioned the rationale for selecting those particular antibiotics in lines 155 to 158.

5. Please remove the CI from the values, e.g., in line 180 instead of 47 (61.84%, CI95: 50.60–71.94), simply write 47 (61.84%)

Response: Thank you. We have now updated that information.

6. There are different types of fruits available in the market. Why were these particular fruits were selected?

Response: Thank you for your query. We selected these fruits because they are available year-round. Most fruits in Bangladesh are seasonal. Some of them have a short shelf life, and some are not suitable for patients. As we selected shops around medical entrances, we considered fruits that are available year-round and are frequently purchased.

7. Line 2412, Moreover, Strong biofilm-forming E. faecalis isolates exhibited higher resistance to certain antibiotics compared to intermediate and non-biofilm formers, explain this observed phenomenon.

Response: Thank you for your suggestion. We have added an explanation in the Discussion section regarding the higher antibiotic resistance observed in strong biofilm-forming E. faecalis isolates. See Line: 296-298

8. Line 252, Furthermore, all the patterns showed resistance to a similar number of antibiotics and classes. Any explanation why similar patterns were observed??

Response: Thank you for your insightful observation. We have removed the line stating that “all the patterns showed resistance to a similar number of antibiotics and classes” as it was redundant and did not add meaningful interpretation.

Reviewer #2:

The manuscript entitlted ‘’ Fresh fruit surface: a potential source for multidrug-resistant biofilm-forming Enterococcus faecalis having potential public health significance’’ presented a well-designed and timely investigation into the occurrence, AMR, biofilm formation and virulence gene profiles of E. faecalis from fresh fruit surfaces in Bangladesh. The study addressed an important gap in food safety and One Health research, particularly in low- and middle income country where surveillance data on antimicrobial-resistant bacteria in fresh produce remain scarce.

The methodology is clearly described, results are logically structured, and discussion is well contextualized within the existing literature. Overall, the manuscript makes a valuable contribution to understanding the public health implication of antimicrobial-resistant enterococci in the food chain.

Besides its novelty and strength, several limitation should be acknowledged more explicitly to make it scientifically sound.

Limitations and Suggestions

1. This study analyzed 76 fruit samples of different types exclusively from Mymensingh city, which is not adequate for an exploratory investigation, and restricts generalizability. Moreover, seasonal variation was not explicitly assessed. Thus, results should be interpreted as region-specific baseline data rather than national prevalence estimates.

Response: We thank the reviewer for this observation. Although our study included 76 fruit samples from Mymensingh city, it provides new insights into antibiotic-resistant, biofilm-forming E. faecalis on fresh fruits in Bangladesh. These results are meant as region-specific baseline data rather than national prevalence estimates, so we selected a few samples, e.g., 76.

2. Only 18 isolates were tested for biofilm formation and 12 isolates for virulence gene profiling, despite 47 PCR-confirmed isolates. The subsampling approach is acceptable for preliminary characterization but limits inferential depth. The authors are suggested to examine all the 47 isolates to make the result scientifically robust.

Response: We thank the reviewer for this valuable suggestion. While we agree that analyzing all 47 PCR-confirmed isolates would strengthen the study, due to limited resources and reagent availability, we were only able to perform biofilm formation assays on 18 isolates and virulence gene profiling on 12 isolates. We have added this as our limitation at the end of the discussion.

3. Biofilm formation was assessed using only the Congo Red Agar (CRA) method, which is qualitative and phenotype dependent. CRA provides rapid screening but may underestimate or overestimate true biofilm biomass.

Response: We thank the reviewer for this important observation. We used the Congo Red Agar (CRA) method as a rapid and simple screening tool for biofilm formation. We acknowledge that CRA is qualitative and phenotype-dependent and may not fully reflect true biofilm biomass; however, it was suitable for preliminary characterization within the resource constraints of our study. We have added this as our limitation at the end of the discussion.

4. Among resistance genes only blaTEM was screened. Key enterococcal resistance genes (e.g. vanA, vanB, ermB tetM, optrA, postA) were not investigated, despite high phenotypic resistance to vancomycin and linezolid. Phenotype-genotype discordance cannot be fully resolved without broader resistance gene profiling.

Response: We thank the reviewer for this suggestion. Due to resource limitations, we screened only for the blaTEM gene, and we acknowledge that including additional resistance genes would provide a more complete genotype-phenotype correlation. We have added this as our limitation at the end of the discussion.

5. The study relies on PCR-based detection rather than whole-genome sequencing. Consequently, clonal relationship, plasmid content, mobile genetic elements, and resistome structure remain unexplored. WGS in recommended for future studies to confirm transmission dynamics and One Health linkages.

Response: We thank the reviewer for this insightful suggestion. In this study, we relied on PCR-based detection due to resource constraints, and we acknowledge that whole-genome sequencing (WGS) would provide deeper insights into clonal relationships, plasmid content, mobile genetic elements, and the overall resistome. Due to the funding crisis, we did not perform WGS. But we plan to incorporate WGS in our future work to better understand transmission dynamics and One Health linkages of E. faecalis from fresh fruits. We have added this as our limitation at the end of the discussion.

Reviewer #3:

Dear Editor,

This manuscript introduces a unique concept in an area that is both intriguing and important for medical research. The topic is timely and is likely to engage the journal's readers. Additionally, the manuscript is well-written, presenting ideas clearly and logically.

Dear Authors,

You are doing excellent work, and this manuscript reflects a great deal of effort and thoughtful research. The study is promising and contributes meaningfully to the field. However, there are a few important points that require further clarification and revision to enhance the overall quality and impact of the manuscript.

Line 44: The Introduction covers several important themes (enterococci biology, public health relevance, fruit contamination, and antibiotic resistance). However, the narrative would benefit from improved coherence and a clearer logical flow. The authors are encouraged to better integrate these topics, explicitly linking Enterococcus faecalis ecology with fruit-associated transmission and public health implications.

Response: Thank you for your suggestion. We have revised the introduction to make it clearer and more coherent.

Line 46: It is preferred to use the following reference to support your updated citations: Alduhaidhawi AHM, AlHuchaimi SN, Al-Mayah TA, Al-Ouqaili MTS, Alkafaas SS, Muthupandian S, Saki M. Prevalence of CRISPR-Cas Systems and Their Possible Association with Antibiotic Resistance in Enterococcus faecalis and Enterococcus faecium Collected from Hospital Wastewater. Infect Drug Resist. 2022 Mar 19;15:1143-1154. Doi: 10.2147/IDR.S358248. PMID: 35340673; PMCID: PMC8942119.

Response: Thank you for your suggestion. We have now updated the line to include a new reference. Reference 1.

Statements suggesting that consumption of fresh fruits may increase the “baseline level of antibiotic resistance” in humans (lines 69–71) are potentially overstated. These claims should be more carefully qualified and supported with stronger evidence or rephrased to avoid causal implications not fully substantiated by current data.

Response: Thank you for your concern and suggestion. We have now modified the sentence. See lines: 71-72

Lines 76–79 repeat nearly identical statements regarding the intrinsic resistance mechanisms of E. faecalis. This redundancy should be eliminated or consolidated into a single, concise description to improve readability and conciseness.

Response: Thank you for your suggestion. We omitted those redundant lines.

Line 76: I recommend that the authors add the following sentences in their introduction using the mentioned studies:

"The increase in multidrug-resistant (MDR) microorganisms triggering infections is growing worldwide and becoming more serious in developing countries."

Response: Thank you. We have added this as suggested. See line: 68-69

In line 76: It is important to use the update and so related reference according to the above comment:

Owaid HA, Al-Ouqaili MTS. Molecular and bacteriological investigations for the co-existence of CRISPR/Cas system and β-lactamases of types extended-spectrum and carbapenemases in Multidrug, extensive drug and Pandrug-Resistant Klebsiella pneumoniae. Saudi J Biol Sci. 2024 Jul;31(7):104022. doi: 10.1016/j.sjbs.2024.104022. Epub 2024 May 19. PMID: 38817398; PMCID: PMC11137337.

Rawaa A. Hussein, Shaymaa H. AL-Kubaisy, Mushtak T.S. Al-Ouqaili. The influence of efflux pump, outer membrane permeability and β-lactamase production on the resistance profile of multi, extensively and pandrug-resistant Klebsiella pneumoniae. Journal of Infection and Public Health, Volume 17, Issue 11, 2024. https://doi.org/10.1016/j.jiph.2024.102544.

\

Response: Thank you for your recommendation. We appreciate it. We used one of the references mentioned to support our claim. However, we cannot use the other references, as they do not align with our claims. See reference 16.

Lines 94–101: The manuscript provides detailed geographic coordinates and fruit types; however, the sampling design is not clearly defined. It remains unclear whether samples were collected using random, convenience, or systematic sampling. The authors should clarify the sampling strategy, inclusion and exclusion criteria, and whether the total sample size (n = 76) was statistically justified.

Response: Thank you for your suggestion. We have now updated the manuscript and included the sampling method. See line: 99-100

Lines 98–101: The number of samples per fruit type is highly uneven (e.g., apples n = 18 vs. dates n = 4). The authors should justify this disparity or discuss its potential impact on prevalence estimates, comparative analyses, and statistical power.

Response: We acknowledge the uneven number of samples per fruit type (e.g., apples n = 18 vs. dates n = 4). This disparity was due to differences in fruit availability and prices, as well as funding limitations. Despite the variation, we aimed to ensure that each fruit type was represented proportionally to its availability in the sampled markets, and this limitation has been discussed in the manuscript regarding its potential impact on prevalence estimates, comparative analyses, and statistical power.

Lines 105–108, 111: The enrichment step involves transferring 100 µL of PBS wash into 1 mL of nutrient broth; however, the rationale for this volume selection is not provided, nor is it clear whether selective enrichment media were considered. Additionally, the term “enriched culture” (line 111) appears to be a typographical error and should be corrected.

Response: Thank you for your suggestion and concern. The 100 µL volume is selected to obtain a representative sample from the PBS wash while maintaining an appropriate inoculum size for bacterial enrichment in 1mL nutrient broth during overnight incubation. This method is well-established in our lab, and we use it frequently in our experiments. After the incubation, we transfer them to the selective media for E. faecalis isolation.

Lines 154–160: The manuscript's claim that nine antibiotics correspond to eight groups requires clarification. Additionally, since rifampin (an ansamycin) is usually used as an adjuvant against Enterococcus spp., a justification for its inclusion in susceptibility testing should be provided.

Response: We thank the reviewer for this comment. The classification has been clarified, and rifampin has been included in accordance with the CLSI 2024 guidelines.

Lines 189–190, 197–198, 248: For prevalence across fruit types and biofilm formation categories, the authors report non-significant differences (p > 0.05) but do not provide test statistics or clarify which tests were used. Including the test type and exact p-values would enhance transparency.

Similarly, differences in antibiotic resistance among biofilm categories are described qualitatively, but statistical comparisons are lacking (Lines 242–248).

Response: Thank you. We have now included the test name in the methods section of the manuscript. We have also noted that the p-value is non-significant at the end of the paragraph. However, these differences were not statistically significant (p>0.05).

Lines 192–196, 205–208, 220–225: Biofilm formation was tested in only 18 out of 47 PCR-positive isolates (Lines 192–196), virulence genes in 12 isolates (Lines 205–208), and antibiotic resistance in 40 isolates (Lines 220–225). The rationale for selecting these subsets is not provided. The authors should justify the sample sizes for these analyses and discuss potential limitations regarding representativeness and statistical power.

Response: Thank you for your concern. Due to funding and resource limitations, we couldn’t test all isolates. However, we have added this as one of our limitations in the end-of-discussions section.

\Lines 212–214, 231–239: Spearman and Pearson correlations between virulence genes and between antibiotics are reported, but the biological interpretation is not clearly explained. The authors should specify the rationale for these correlations and discuss potential confounding factors. Some correlations are repeated or redundant (Lines 235–237: Linezolid and Vancomycin are reported twice). The manuscript should remove duplicates to avoid confusion.

Response: Thank you for the comment. We have revised the manuscript to include a clear biological interpretation of the Spearman and Pearson correlations in the Discussion section, explaining the rationale for analyzing co-occurrence patterns among virulence genes and antibiotic resistances, as well as potential confounding factors. Additionally, all duplicate correlations, including the repeated Linezolid–Vancomycin entry, have been removed to avoid confusion.

Lines 250–253: The MDR prevalence and MAR index are reported, but methods for calculating MAR for each isolate are not linked to the results clearly. The authors should p

Attachment Submitted filename: Reviwer Response.docx

# Peer review: Decision Letter 1

**DOI:** 10.1371/journal.pone.0355410.r003

**Authors:** Leonard Ighodalo Uzairue

28 May 2026

PONE-D-25-60195R1

Fresh fruit surface: a potential source for multidrug-resistant biofilm-forming Enterococcus faecalis having potential public health significance

PLOS One

Dear Dr. Rahman,

Thank you for submitting your manuscript to PLOS ONE. After careful consideration, we feel that it has merit but does not fully meet PLOS ONE’s publication criteria as it currently stands. Therefore, we invite you to submit a revised version of the manuscript that addresses the points raised during the review process.

Please submit your revised manuscript by Jul 12 2026 11:59PM. If you will need more time than this to complete your revisions, please reply to this message or contact the journal office at plosone@plos.org. When you're ready to submit your revision, log on to https://www.editorialmanager.com/pone/ and select the 'Submissions Needing Revision' folder to locate your manuscript file.

Please include the following items when submitting your revised manuscript:

- A letter that responds to each point raised by the academic editor and reviewer(s). You should upload this letter as a separate file labeled 'Response to Reviewers'.
- A marked-up copy of your manuscript that highlights changes made to the original version. You should upload this as a separate file labeled 'Revised Manuscript with Track Changes'.
- An unmarked version of your revised paper without tracked changes. You should upload this as a separate file labeled 'Manuscript'.

If you would like to make changes to your financial disclosure, please include your updated statement in your cover letter. Guidelines for resubmitting your figure files are available below the reviewer comments at the end of this letter.

If applicable, we recommend that you deposit your laboratory protocols in protocols.io to enhance the reproducibility of your results. Protocols.io assigns your protocol its own identifier (DOI) so that it can be cited independently in the future. For instructions see: https://journals.plos.org/plosone/s/submission-guidelines#loc-laboratory-protocols. Additionally, PLOS ONE offers an option for publishing peer-reviewed Lab Protocol articles, which describe protocols hosted on protocols.io. Read more information on sharing protocols at https://plos.org/protocols?utm_medium=editorial-email&utm_source=authorletters&utm_campaign=protocols.

As the corresponding author, your ORCID iD is verified in the submission system and will appear in the published article. PLOS supports the use of ORCID, and we encourage all coauthors to register for an ORCID iD and use it as well. Please encourage your coauthors to verify their ORCID iD within the submission system before final acceptance, as unverified ORCID iDs will not appear in the published article. *Only*  the individual author can complete the verification step; PLOS staff *cannot*  verify ORCID iDs on behalf of authors.

We look forward to receiving your revised manuscript.

Kind regards,

Leonard Ighodalo Uzairue, PhD

Academic Editor

PLOS One

Journal Requirements:

If the reviewer comments include a recommendation to cite specific previously published works, please review and evaluate these publications to determine whether they are relevant and should be cited. There is no requirement to cite these works unless the editor has indicated otherwise.

[Note: HTML markup is below. Please do not edit.]

Reviewers' comments:

Reviewer's Responses to Questions

**Comments to the Author**

1. If the authors have adequately addressed your comments raised in a previous round of review and you feel that this manuscript is now acceptable for publication, you may indicate that here to bypass the “Comments to the Author” section, enter your conflict of interest statement in the “Confidential to Editor” section, and submit your "Accept" recommendation.

Reviewer #6: (No Response)

**********

2. Is the manuscript technically sound, and do the data support the conclusions?

The manuscript must describe a technically sound piece of scientific research with data that supports the conclusions. Experiments must have been conducted rigorously, with appropriate controls, replication, and sample sizes. The conclusions must be drawn appropriately based on the data presented.

Reviewer #6: Yes

**********

3. Has the statistical analysis been performed appropriately and rigorously?

Reviewer #6: Yes

**********

4. Have the authors made all data underlying the findings in their manuscript fully available?

The PLOS Data policy requires authors to make all data underlying the findings described in their manuscript fully available without restriction, with rare exception (please refer to the Data Availability Statement in the manuscript PDF file). The data should be provided as part of the manuscript or its supporting information, or deposited to a public repository. For example, in addition to summary statistics, the data points behind means, medians and variance measures should be available. If there are restrictions on publicly sharing data—e.g. participant privacy or use of data from a third party—those must be specified.

Reviewer #6: Yes

**********

5. Is the manuscript presented in an intelligible fashion and written in standard English?

PLOS ONE does not copyedit accepted manuscripts, so the language in submitted articles must be clear, correct, and unambiguous. Any typographical or grammatical errors should be corrected at revision, so please note any specific errors here.

Reviewer #6: Yes

**********

6. Review Comments to the Author

Please use the space provided to explain your answers to the questions above. You may also include additional comments for the author, including concerns about dual publication, research ethics, or publication ethics. (Please upload your review as an attachment if it exceeds 20,000 characters)

Reviewer #6: Thank you for your careful revision. The manuscript has improved substantially in clarity, organization, and transparency. The study is relevant and contributes useful baseline data on antimicrobial-resistant Enterococcus faecalis in fresh fruits.

However, the following issues remain that need to be addressed

1. The study confirms 47 isolates of E. faecalis, yet:

• Biofilm analysis: 18 isolates

• Virulence genes: 12 isolates

This represents a significant reduction in analytical coverage.

While you have acknowledged this limitation, the manuscript still states that virulence genes and biofilm traits are “widely distributed”, which is not fully supported by the subsampled data.

To address this the author should:

• Rephrase conclusions to indicate findings are preliminary or indicative

• Avoid generalizing to all isolates

• Explicitly state that prevalence may be over- or under-estimated

2. The reported resistance levels: Vancomycin: 47.5% and Linezolid: 47.5% are unexpectedly high for food-derived isolates and require stronger caution. Although you mention CLSI limitations, this is not sufficiently emphasized.

The Critical issue here is that: Disk diffusion is not the preferred method for these antibiotics in enterococci, and may lead to inaccurate classification.

To address this the author is Required to:

• Add a strong statement that:

o These results may overestimate resistance

o MIC-based confirmation is required

• Avoid implying confirmed presence of:

o Vancomycin-resistant enterococci (VRE)

o Linezolid-resistant strains

• Tone down conclusions accordingly, especially in the Conclusion section

3. The manuscript reports correlation values (e.g., ρ = 1.000) that are statistically questionable, especially given small sample sizes. The concerns here are:

• Likely artefacts of small sample size

• No correction for multiple comparisons

• Limited biological interpretability

The required revisions in this circumstances is for the author to:

• Clearly state that:

o Correlation analysis is exploratory

o Results are hypothesis-generating only

• Add a statement on risk of Type I error

• Interpret correlations cautiously or move to supplementary material

4. The study includes: 76 fruit samples from a single city (Mymensingh), Uneven distribution across fruit types, This limits generalizability.

The author should:

• Clearly describe sampling as:

o “localized” or “site-specific”

• Emphasize findings as:

o baseline data, not national estimates

• Avoid general statements about broader populations

5. The manuscript suggests that fruit surfaces pose a “significant risk of transmission to humans”

This statement is too strong given that there is:

• No exposure assessment

• No transmission pathway analysis

• No genomic linkage to human infections

Required revisions:

• Replace with cautious phrasing such as:

o “may pose a potential risk”

o “could contribute to exposure”

• Avoid causal or definitive public health claims

6. The following minor improvements in language and presentation are also recommended:

a. The manuscript Language and Grammar has improved but still contains:

o Typographical errors

o Awkward phrasing

Professional language editing is recommended

b. MAR index formula is included but should be clearly linked to results

c. Clarify how isolates were selected for subsampling (randomization process)

d. CRA is correctly described

e. However, emphasize more clearly that it is:

o qualitative

o less precise than quantitative assays

f. Ensure all figures and tables:

o Include sample sizes (n)

o Have self-explanatory captions

g. Ensure consistency in formatting

**********

7. PLOS authors have the option to publish the peer review history of their article (what does this mean?). If published, this will include your full peer review and any attached files.

If you choose “no”, your identity will remain anonymous but your review may still be made public.

**Do you want your identity to be public for this peer review?**  For information about this choice, including consent withdrawal, please see our Privacy Policy.

Reviewer #6: **Yes:**  Lawan Adamu

**********

[NOTE: If reviewer comments were submitted as an attachment file, they will be attached to this email and accessible via the submission site. Please log into your account, locate the manuscript record, and check for the action link "View Attachments". If this link does not appear, there are no attachment files.]

To ensure your figures meet our technical requirements, please review our figure guidelines: https://journals.plos.org/plosone/s/figures

You may also use PLOS’s free figure tool, NAAS, to help you prepare publication quality figures: https://journals.plos.org/plosone/s/figures#loc-tools-for-figure-preparation.

NAAS will assess whether your figures meet our technical requirements by comparing each figure against our figure specifications.

# Author comment: Author response to Decision Letter 2

**DOI:** 10.1371/journal.pone.0355410.r004

29 May 2026

6. Review Comments to the Author

Please use the space provided to explain your answers to the questions above. You may also include additional comments for the author, including concerns about dual publication, research ethics, or publication ethics. (Please upload your review as an attachment if it exceeds 20,000 characters)

Reviewer #6: Thank you for your careful revision. The manuscript has improved substantially in clarity, organization, and transparency. The study is relevant and contributes useful baseline data on antimicrobial-resistant Enterococcus faecalis in fresh fruits.

However, the following issues remain that need to be addressed

1. The study confirms 47 isolates of E. faecalis, yet:

• Biofilm analysis: 18 isolates

• Virulence genes: 12 isolates

This represents a significant reduction in analytical coverage.

While you have acknowledged this limitation, the manuscript still states that virulence genes and biofilm traits are “widely distributed”, which is not fully supported by the subsampled data.

To address this the author should:

• Rephrase conclusions to indicate findings are preliminary or indicative

• Avoid generalizing to all isolates

• Explicitly state that prevalence may be over- or under-estimated

Response: Thank you so much for your suggestions. We have rephrased our claims and updated the manuscript. See line: 386-388

2. The reported resistance levels: Vancomycin: 47.5% and Linezolid: 47.5% are unexpectedly high for food-derived isolates and require stronger caution. Although you mention CLSI limitations, this is not sufficiently emphasized.

The Critical issue here is that: Disk diffusion is not the preferred method for these antibiotics in enterococci, and may lead to inaccurate classification.

To address this the author is Required to:

• Add a strong statement that:

o These results may overestimate resistance

o MIC-based confirmation is required

• Avoid implying confirmed presence of:

o Vancomycin-resistant enterococci (VRE)

o Linezolid-resistant strains

• Tone down conclusions accordingly, especially in the Conclusion section

Response: Thank you for your suggestions. Now, we have modified and rephrased the statements to tone them down. See line: 349-350

3. The manuscript reports correlation values (e.g., ρ = 1.000) that are statistically questionable, especially given small sample sizes. The concerns here are:

• Likely artefacts of small sample size

• No correction for multiple comparisons

• Limited biological interpretability

The required revisions in this circumstances is for the author to:

• Clearly state that:

o Correlation analysis is exploratory

o Results are hypothesis-generating only

• Add a statement on risk of Type I error

• Interpret correlations cautiously or move to supplementary material

Response: Thank you for your feedback. We have updated the manuscript. See line: 365-370

4. The study includes: 76 fruit samples from a single city (Mymensingh), Uneven distribution across fruit types, This limits generalizability.

The author should:

• Clearly describe sampling as:

o “localized” or “site-specific”

• Emphasize findings as:

o baseline data, not national estimates

• Avoid general statements about broader populations

Response: Thank you. We have updated the manuscript. See line: 399

5. The manuscript suggests that fruit surfaces pose a “significant risk of transmission to humans”

This statement is too strong given that there is:

• No exposure assessment

• No transmission pathway analysis

• No genomic linkage to human infections

Required revisions:

• Replace with cautious phrasing such as:

o “may pose a potential risk”

o “could contribute to exposure”

• Avoid causal or definitive public health claims

Response: Thank you for your suggestions. Now we have rephrased the statement. See line: 406

6. The following minor improvements in language and presentation are also recommended:

a. The manuscript Language and Grammar has improved but still contains

o Typographical errors

o Awkward phrasing

Professional language editing is recommended

b. MAR index formula is included but should be clearly linked to results

c. Clarify how isolates were selected for subsampling (randomization process)

d. CRA is correctly described

e. However, emphasize more clearly that it is:

o qualitative

o less precise than quantitative assays

Response: Thank you for your suggestions. We have updated the manuscript.

f. Ensure all figures and tables:

o Include sample sizes (n)

o Have self-explanatory captions

g. Ensure consistency in formatting

Response: Thank you for your suggestions. We have updated the manuscript.

Attachment Submitted filename: Reviwer_Response_auresp_2.docx

# Editor report: Decision Letter 2

**DOI:** 10.1371/journal.pone.0355410.r005

**Authors:** Guadalupe Virginia Nevárez-Moorillón

21 Jul 2026

Fresh fruit surface: a potential source for multidrug-resistant biofilm-forming Enterococcus faecalis having potential public health significance

PONE-D-25-60195R2

Dear Dr. Rahman,

We’re pleased to inform you that your manuscript has been judged scientifically suitable for publication and will be formally accepted for publication once it meets all outstanding technical requirements.

Within one week, you’ll receive an e-mail detailing the required amendments. When these have been addressed, you’ll receive a formal acceptance letter and your manuscript will be scheduled for publication.

An invoice will be generated when your article is formally accepted. Please note, if your institution has a publishing partnership with PLOS and your article meets the relevant criteria, all or part of your publication costs will be covered. Please make sure your user information is up-to-date by logging into Editorial Manager at Editorial Manager® and clicking the ‘Update My Information' link at the top of the page. For questions related to billing, please contact billing support.

If your institution or institutions have a press office, please notify them about your upcoming paper to help maximize its impact. If they’ll be preparing press materials, please inform our press team as soon as possible -- no later than 48 hours after receiving the formal acceptance. Your manuscript will remain under strict press embargo until 2 pm Eastern Time on the date of publication. For more information, please contact onepress@plos.org.

Kind regards,

Guadalupe Virginia Nevárez-Moorillón, Ph.D.

Academic Editor

PLOS One

Additional Editor Comments (optional):

Reviewers' comments:

# Editor report: Acceptance letter

**DOI:** 10.1371/journal.pone.0355410.r006

**Authors:** Guadalupe Virginia Nevárez-Moorillón

PONE-D-25-60195R2

PLOS One

Dear Dr. Rahman,

I'm pleased to inform you that your manuscript has been deemed suitable for publication in PLOS One. Congratulations! Your manuscript is now being handed over to our production team.

At this stage, our production department will prepare your paper for publication. This includes ensuring the following:

* All references, tables, and figures are properly cited

* All relevant supporting information is included in the manuscript submission,

* There are no issues that prevent the paper from being properly typeset

You will receive further instructions from the production team, including instructions on how to review your proof when it is ready. Please keep in mind that we are working through a large volume of accepted articles, so please give us a few days to review your paper and let you know the next and final steps.

Lastly, if your institution or institutions have a press office, please let them know about your upcoming paper now to help maximize its impact. If they'll be preparing press materials, please inform our press team within the next 48 hours. Your manuscript will remain under strict press embargo until 2 pm Eastern Time on the date of publication. For more information, please contact onepress@plos.org.

You will receive an invoice from PLOS for your publication fee after your manuscript has reached the completed accept phase. If you receive an email requesting payment before acceptance or for any other service, this may be a phishing scheme. Learn how to identify phishing emails and protect your accounts at https://explore.plos.org/phishing.

If we can help with anything else, please email us at customercare@plos.org.

Thank you for submitting your work to PLOS ONE and supporting open access.

Kind regards,

PLOS ONE Editorial Office Staff

on behalf of

Dr. Guadalupe Virginia Nevárez-Moorillón

Academic Editor

PLOS One
